# Implementation and validation of an in-house combined fluorescein/media-fill test to qualify radiopharmacy operators

**DOI:** 10.1186/s41181-020-00117-6

**Published:** 2021-01-07

**Authors:** Cyril Fersing, Emmanuel Deshayes, Sarah Langlet, Laurence Calas, Vincent Lisowski, Pierre Olivier Kotzki

**Affiliations:** 1 Department of Nuclear Medicine, Montpellier Cancer Institute (ICM), University of Montpellier, 208 avenue des apothicaires, 34298 Montpellier Cedex 5, France; 2 Institut des Biomolécules Max Mousseron, UMR 5247, CNRS, Université de Montpellier, ENSCM, UFR des Sciences Pharmaceutiques et Biologiques, Montpellier Cedex, France; 3 University of Montpellier, Institut de Recherche en Cancérologie de Montpellier (IRCM), INSERM U1194, Montpellier Cancer Institute (ICM), Montpellier, France; 4 Department of Nuclear Medicine, Saint-Jean Hospital, Perpignan, France; 5 Quality Control Laboratory, University Hospital of Montpellier, Montpellier, France

**Keywords:** Media-fill test, Fluorescein, Good radiopharmacy practices, Sterile compounding, Operators qualification

## Abstract

**Background:**

The purpose of this work was to design, validate and implement a media-fill test combined with fluorescein (MFT-F) for the specific qualification and training of radiopharmacy operators, in accordance with United States Pharmacopeia General Chapter 797 and European Good Manufacturing Practices. MFT-F was embedded in the quality management system of our radiopharmacy unit. Its validation involved fluorescein concentration choice, media growth promotion test and evaluation protocol controls (with or without intentional aseptic mistakes). Each operator was evaluated following a three-part evaluation form. Evaluation criteria related to garbing and hygiene, fluorescent contamination and bacteriological contamination (pre- and post-evaluation environment controls and MFT-F samples). Combined MFT-F allowed the assessment of aseptic compounding skills and non-contamination of the working area through a single evaluation. It was also designed to fit the constraints of radiopharmacy common practice related to radiation protection equipment and to the small volumes handled.

**Results:**

A 0.01% fluorescein concentration was chosen to prepare MFT-F. Addition of fluorescein in the culture medium did not jeopardize its growth properties according to growth promotion test. Eleven operators were evaluated and carried out 3 MFT-F over 3 successive days. Pre- and post-evaluation bacteriological controls of every session showed no CFU of microbiological contaminant above 5. All operators validated the garbing and hygiene evaluation, with an average score of 92.7%. All operators validated the fluorescent contamination evaluation, with an average score of 29.4 out of 30. None of the MFT-F samples showed any visible bacterial growth after incubation.

**Conclusions:**

Combined MFT-F, as a part of a comprehensive sterile compounding training program, appeared as a convenient and promising tool to increase both the sterile compounding safety and awareness of radioactive contamination in radiopharmacy.

**Supplementary Information:**

The online version contains supplementary material available at 10.1186/s41181-020-00117-6.

## Background

As a core field in the functioning of a Nuclear Medicine unit, radiopharmacy activities essentially consist of the preparation of radiopharmaceuticals for intravenous administration. Although some radiopharmaceuticals are manufactured industrially in a ready-to-use-form, a significant proportion of these particular drugs is prepared onsite, in the radiopharmacy unit of healthcare establishments (Decristoforo and Patt, 2017). To carry out these preparations, the handling of unsealed radioactivity sources is required, involving both radiation protection and aseptic technique concerns. Indeed, radiopharmaceutical preparations should comply with sterility requirements for parenterals (Decristoforo et al. 2014) while ensuring sufficient radiation protection for operators and the absence of environmental radiocontamination (Biechlin et al. 2007). Because of the risks underlying these requirements (Staes et al. 2013; Shehab et al. 2018), hospital activities involving preparations, such as radiopharmacy but also oncology pharmacy or parenteral nutrition compounding, must be associated with efficient quality management systems and appropriate training methods (Myers, 2013). Thus, process control and both initial and periodic qualification of operators are key elements for securing preparation activities (Mullarkey, 2009), tending to be included within the scope of quality assurance. To date, the sterile compounding training program in our Nuclear Medicine department included (a) didactic classroom instructions provided by a qualified radiopharmacist, (b) evaluation of professional practices with observed skill assessment of aseptic handling, and (c) direct skill-focused validation of the prior learning at the radiopharmacy laboratory, with evaluation by the radiopharmacist. Thus, to better meet both EU GMP (EudraLex, 2015) and United States Pharmacopeia (USP) General Chapter 797 on quality standards for compounding sterile preparations (USP, 2017), a revision of our training program has been considered, including the implementation of a media-fill test (MFT).

A MFT is the performance of aseptic manipulations in real working conditions, using a sterile microbiological growth medium like tryptic soy broth (TSB) instead of the usual preparation solution (e.g. sodium pertechnetate eluate in radiopharmacy). In addition to being useful for aseptic processes qualification (Kawamura, 2002), MFTs play a valuable role in operators training (Nemec et al. 2016). To be initially qualified in aseptic compounding, operators should perform 3 MFTs on 3 separate days. An annual requalification by performing 1 MFT is subsequently recommended. Several ready-to-use MFT kits containing empty sterile vials and culture medium-filled vials, bags or ampuls are commercially available. However, these classic MFTs don’t allow easy assessment of a work environment contamination during the test, which is an essential concern in radiopharmacy practice. Indeed, work environment non-contamination is a way to reduce radiation exposure of personnel during preparation (Heller, 1996) and limit the risk of contamination spreading (Tazrart et al. 2013; Covens et al. 2012). This is a particularly crucial point for radioelements with a long physical half-life (e.g. ^111^In). Noteworthy, commercially available MFT kits are also hardly compatible with some radiation protection elements commonly used in radiopharmacy, such as shielded vial protections, and with the small volumes usually handled for radiopharmaceuticals preparations.

The use of a fluorescein solution to mimic the hazardous substance to be handled is an attractive test to evaluate the safety and absence of leakage during transfers (Dussart et al. 2008; Segner et al. 2017), especially when closed-system transfer devices are involved (Favier et al. 2012; Garrigue et al. 2016). It could therefore be possible to carry out an MFT to assess aseptic compounding skills, followed by a fluorescein test to check the non-contamination of the working area. However, operators evaluation through a single test would be much more consistent. As there is, to our knowledge, no commercial kit presented as a combined media-fill and fluorescein test (MFT-F), our objective was to design a comprehensive MFT-F protocol, from its production to its implementation in our radiopharmacy unit as a compounding training to ensure compliance with current standards and reduce staff practice variability. With the establishment of a practical methodology to simultaneously evaluate these two aspects, this « in house » MFT-F was intended as a part of our general sterile compounding instruction for operators, also considering garbing and hygiene practices. One of the main interests of this test is to take into account the radioprotection constraints systematically applied in standard radiopharmacy practice.

## Methods

### Generalities

MFT-F were performed in the same radiopharmaceutical preparation laboratory, in sessions consecutive to daily routine, in the radiopharmacy unit of the Nuclear Medicine Department of Montpellier Cancer Institute, France. All commonly used products (syringes, needles, vials …) were sterile and pyrogen-free. Incubation of samples produced during the development and implementation of the MFT-F was carried out in an internally qualified oven monitored by an external temperature sensor and located in a secured facility.

### Choice of fluorescein concentration

The fluorescence yield of fluorescein varies according to concentration, causing quenching at too high concentrations. Maximum fluorescence is reached at 0.002% fluorescein in aqueous solution while fluorescence decreases for higher concentrations (Amalric, 1972). In order to verify this parameter for a fluorescein solution in TSB, successive dilutions were prepared in glass test tubes (10%, 5%, 2.5%, 1%, 0.5%, 0.05%, 0.01% and 0.002%) and were visually examined under ultraviolet light. From each of these solutions, a 2 μL drop was collected, placed on a sterile field to simulate a splashed drop, and visually examined under UV light.

### Tryptic soy broth-fluorescein (TSB-F) vials preparation

**Fig. 1:**
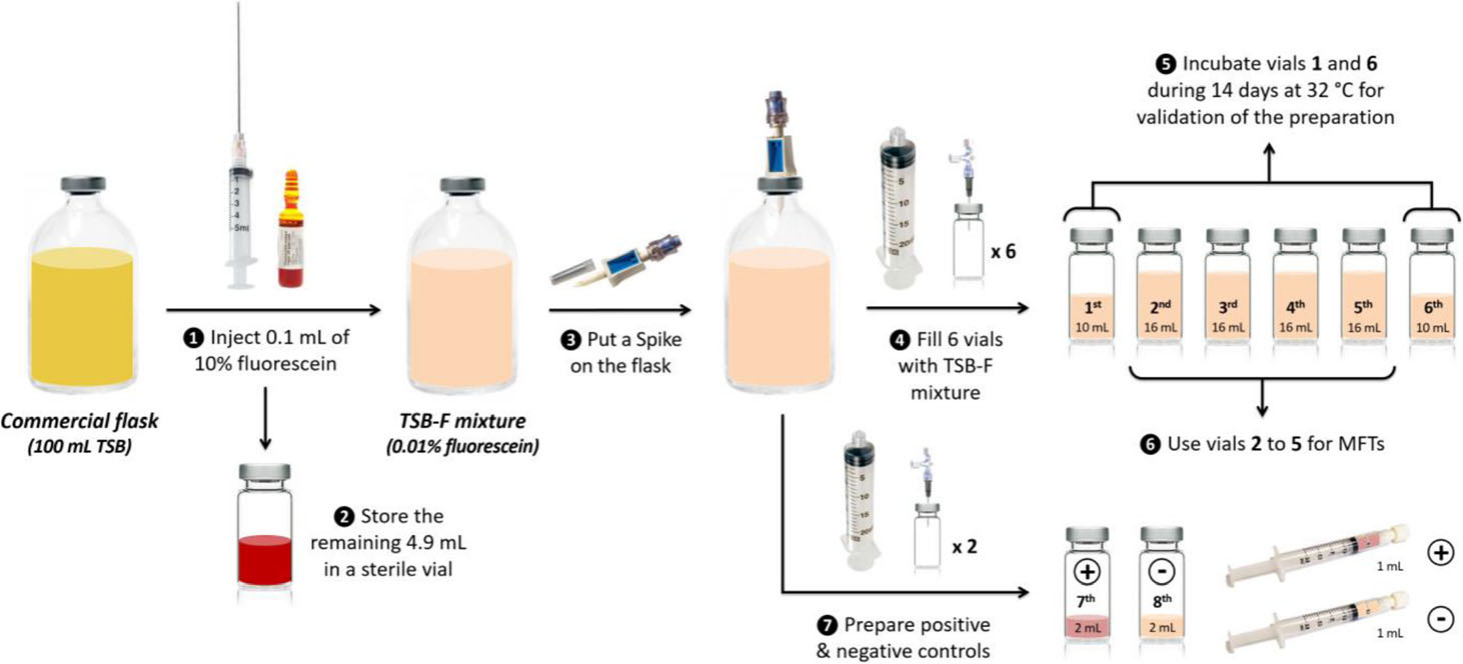
preparation of tsb-f vials

This step was carried out inside a class II laminar airflow hood placed in a class D controlled area. Vial preparation procedure is shown in Fig. 1. First, 100 μL of fluorescein 10% solution (pharmaceutical grade) were aseptically added to a 100 mL sealed sterile TSB flask (VWR). Then, 6 elution vials (TC-ELU-5®, CIS bio international) were filled with TSB-F. Vials 1 and 6 (10 mL TSB-F each) were incubated for aseptic validation of the manipulation and vials 2 to 5 (16 mL TSB-F each) were stored at 4–8 °C not more than 10 days, before use during MFT-F. Vials 7 and 8 (2 mL TSB-F each) and two 3 mL syringes (1 mL TSB-F each) capped with a luer-lock stopping plug were prepared as positive and negative controls. Positive controls were spoiled with a few microliters of contaminated TSB medium. Detailed TSB-F preparation protocol is available in Supplementary data S1.

### Media growth promotion test

To confirm the ability of TSB to support bacterial growth in the presence of 0.01% fluorescein, a verification procedure was undertaken following USP 71 (USP, 2018) and *European Pharmacopoea* recommendations (Council of Europe, 2018, 2018). The test used a lyophilized *Bacillus subtilis spizizenii* preparation kit (EZ-ACCU SHOT™ ATCC®, Microbiologics Corporation). Inside a class II laminar airflow hood, 3 vials with 2 mL TSB-F were each seeded with 140 μL of a reconstituted 710 CFU/mL *B. subtilis* suspension. As a positive control, a 2 mL TSB vial without fluorescein was seeded the same way. A 2 mL TSB vial and a 2 mL TSB-F vial were used as negative controls. These 7 units were incubated during 3 days at 32 °C and visually examined.

### Kit preparation

To facilitate the start-up of an MFT, the single-use equipment necessary for an operator evaluation was priorly gathered in a hermetic plastic bag. A complete kit contained four 5 mL syringes, six 3 mL syringes, four sterile 15 mL elution vials labeled A to D, six 23G 25 mm needles, four 22G 30 mm needles, five 23G 60 mm needles, five luer-lok shutters with injection site, sterile non-woven swabs and one sterile disposable drape.

### Pre- and post-evaluation bacteriological controls

**Fig. 2:**
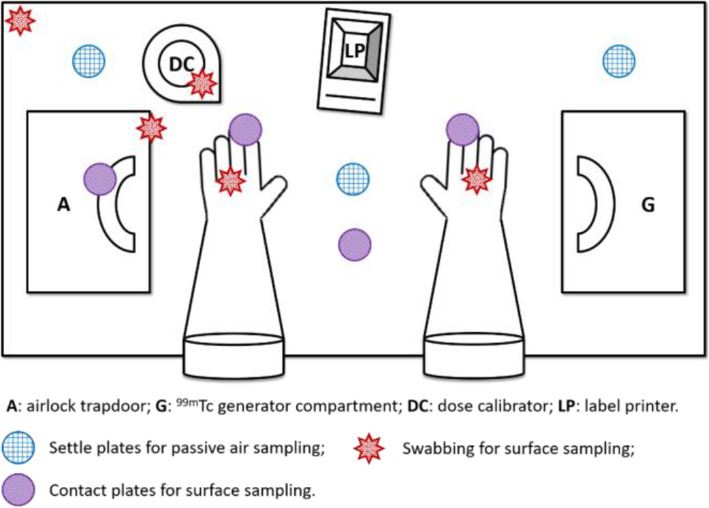
cartographic representation of the lead-shielded cell working area showing the locations determined for pre- and post-evaluation bacteriological controls

Control of sterility throughout the whole aseptic handling process had to be validated before the introduction of a MFT. MFT were carried out in a shielded laminar flow shielded cell (ELIZA Series, Comecer), operational and certified class A, placed in the radiopharmaceutical preparation laboratory (class D controlled area). This shielded cell was equipped with an internal 257 nm UV lamp. Before and after each MFT session, surface sampling of two critical planar surfaces (near the handle of the airlock trapdoor and preparation area in front of the operator) was operated by a qualified radiopharmacist, using contact plates. Surface sampling of five nonplanar surfaces (seal of the airlock trapdoor, dipper in the dose calibrator, left hand corner of the working area and interdigital spaces of both gloves) was also carried out using sterile swabs for dry collection incubated in tubes with TSB. Fingerprints from fingertips and thumbs of both gloves were performed by pressing on contact plates for 10 s. After each MFT session, passive air sampling inside the shielded cell was performed by positioning settle plates at 3 predetermined areas (preparation area in front of the operator, left and right hand corner of the working area) during 4 h. Bacteriological samples inside the shielded cell are summarized in Fig. 2. Surface sampling in the class D controlled area was undertaken weekly throughout the MFT campaign at five predetermined surfaces (two workbenches, computer keyboard, external trapdoor of the airlock and above a mobile material cabinet). Passive air sampling in the radiopharmaceutical preparation laboratory was performed by positioning settle plates at two predetermined areas (workbenches).

### Operator evaluation form

**Fig. 3:**
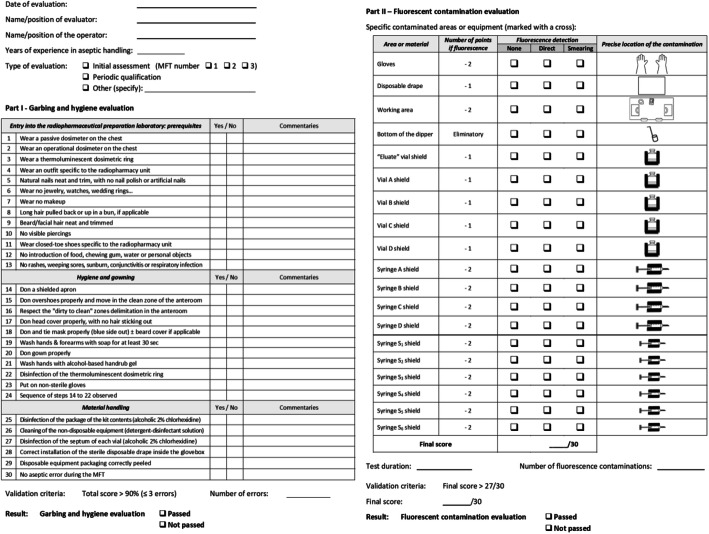
first and second parts of the operator evaluation form

To qualify the operators, a three-part validation form was established, both for initial and periodic evaluation (Fig. 3). First section, inspired by our internal procedures, gathered evaluation criteria following FGMP and USP 797 garbing and hygiene recommendations (EudraLed n.d.; USP< 797> n.d.) through 30 items. Radiation protection recommendations were also evaluated (Elsinga et al. 2010). A negative answer should be considered as an error, a total score > 90% (≤ 3 errors) being required to validate this section. Second part of this form provided for the counting and characterization of fluorescein-contaminated areas or devices, with a negative scoring system to rate operators according to the number and critical nature of contaminations. Final scores equal to or superior than 27/30 (90%) are expected to pass each part of the evaluation. Third part is reserved for the daily monitoring of incubated units. Operator evaluation form is available in Supplementary data S2.

### Operator evaluation protocol

Operators were individually trained and evaluated by one and only qualified radiopharmacist, after explaining the test issues and process. Initial practical training was in accordance with FGMP and USP guidelines for sterile preparations in hospital pharmacies. MFT sessions were scheduled after working days, without cleaning the shielded cell preparation area before the test. Two operators were evaluated successively during each session. Operators were first evaluated on garbing and hygiene common practice before entering the preparation laboratory (see Fig. 3). Once in the laboratory, operators opened an evaluation kit and carefully disinfected the package of each medical device and the septum of each vial with 70% isopropyl alcohol before entering the shielded cell. In the same way, non-disposable equipment was cleaned using a detergent-disinfectant solution. This equipment included five vial shields identified “Eluate” and from A to D, four 5 mL syringe shields identified from A to D, six 3 mL syringe shields identified from 1 to 6 and one 30 cm long forceps usually used to safely handle radioactivity-containing vials. A single vial of TSB-F mixture was used during a test.

**Fig. 4:**
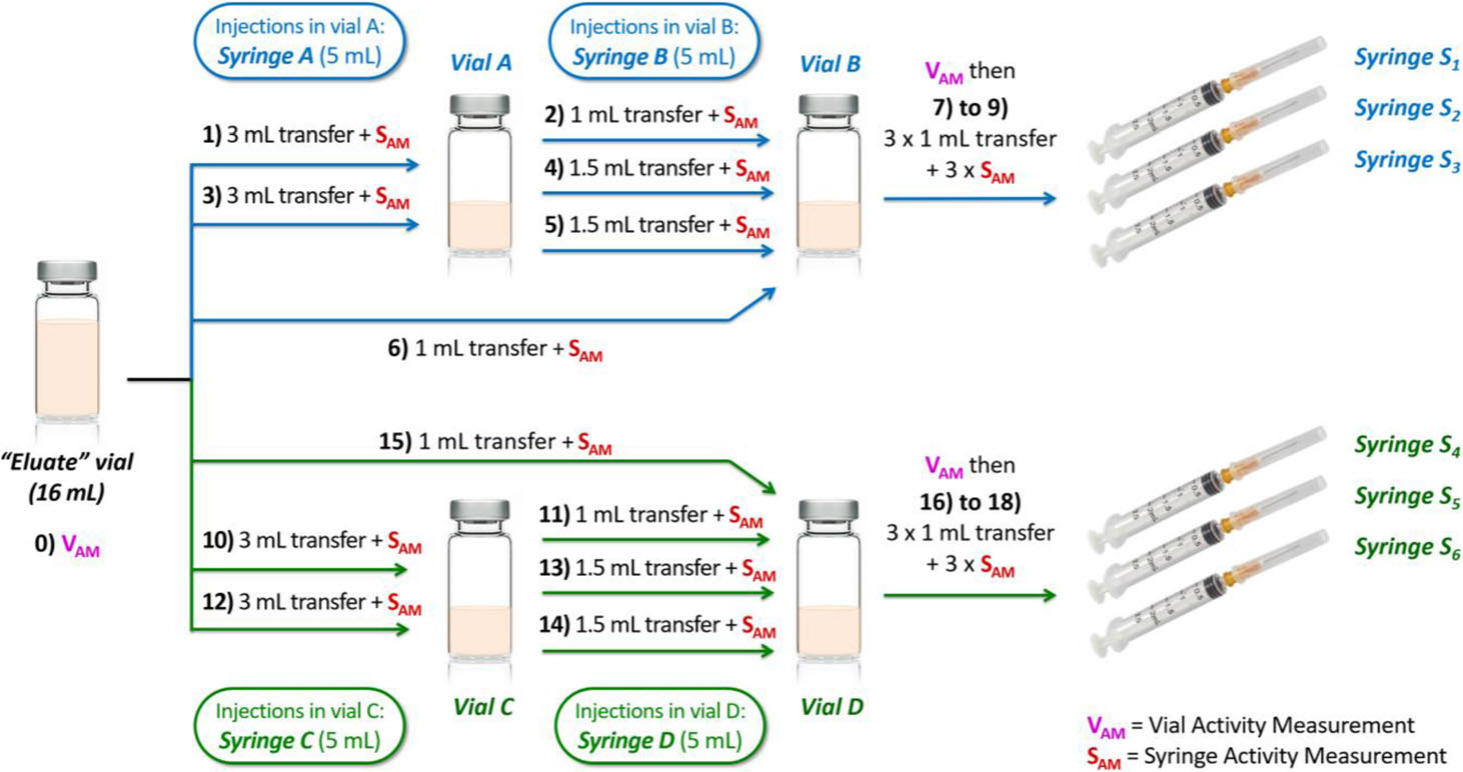
mft-f operating procedure

Before starting the test, absence of fluorescent traces in the working area and on the gloves was checked using the shielded cell UV lamp. Once all the equipment was in the shielded cell, the operator began the experimental filling operations as illustrated in Fig. 4. A first sequential 2-vials preparation simulation was started, with TSB-F transfers from “Eluate” vial to vial A or vial B and from vial A to vial B. For every transfer, syringe or vial activity measurement was simulated by placing it in the well of the dose calibrator. At the end of the transfer sequence, 3 simulated patient doses were prepared. A second identical preparation sequence was repeated with the same “Eluate” vial and 2 new preparation vials. Detailed MFT-F protocol is available in Supplementary data S3.

### Incubation and reading of pre- and post-evaluation bacteriological controls results

Contact plates, settle plates and sterile swabs in TSB tubes were placed for incubation 7 days at room temperature (22 °C ± 2 °C) to promote the development of environmental germs. Then, the same units were incubated 7 more days at 32 °C under 5% CO_2_ to promote the development of bacterial contaminants of human origin. Samples were monitored daily by counting the total number of discrete colonies on each plate and checking the turbidity of TSB tubes. Positive and negative control agar plates and TSB tubes were prepared and incubated simultaneously.

### Incubation and reading of operator evaluation results

At the end of a MFT, all the equipment used for the evaluation was taken out of the glove box and was carefully examined in the dark by the radiopharmacist, under UV light. Absence of fluorescent traces in the working area and on the gloves was checked using the shielded cell UV lamp. A smearing was then carried out on all the surfaces previously observed (inside the shielded cell and on equipment) using a compress slightly soaked in NaCl 0.9%. The compress was carefully examined under UV light to uncover fluorescent traces and improve the detection sensitivity for micro-projections. The limit of detection on the compress was previously assessed as ≤1 μL TSB-F. Fluorescein contaminations are reported on the Operator evaluation form (see Fig. 3). Before incubation, internal surfaces of vial A to D, “Eluate vial” and syringes S_1_ to S_6_ were brought into contact with TSB-F mixture by inversion. For syringes, the piston seal was positioned on the 2 mL graduation to allow aerobic conditions. Each unit was incubated 7 days at room temperature then 7 more days at 32 °C under 5% CO_2_. Positive and negative controls were incubated similarly and simultaneously. All units were visually inspected daily by the unaided eye, with gentle mixing.

### Validation of the operator evaluation protocol

MFT-F controls were done by a single trained radiopharmacist. A first series of 3 MFT-F following the previously described protocol was achieved using good aseptic technique. Then, a second series of 3 MFT-F was achieved with intentional aseptic mistakes. These included no prior disinfection of surfaces, equipment or gloves, and intentional finger dab on the septum and on the luer-lok stoppers of vials. Incubation and daily observation were conducted similarly to the methods used during operators assessment.

### Cost study

The cost of making and applying one MFT-F was estimated, considering the price of the equipment used and the time spent by the radiopharmacist. This estimated cost was then compared to the commercially available MFT kits.

## Results

A 0.01% fluorescein concentration in TSB was chosen to prepare TSB-F vials because of its most intense fluorescence among the tested dilutions. Through 10 preparation sessions, 40 TSB-F vials were made, with no bacterial growth in any aseptic validation vial. A total of 33 kits were prepared to ensure all qualification sessions. Operator evaluation protocol validation showed that none of the incubated units from the 3 MFT-F achieved with good aseptic technique grew positive. In contrast, at least 7 of the 11 incubated units from each 3 MFT-F achieved with intentional aseptic mistakes grew positive. The growth promotion test showed a clear bacterial growth in the 3 TSB-F vials and in positive controls while no turbidity was observed in the negative control. This result demonstrated that 0.01% fluorescein in TSB did not jeopardize the growth properties of the culture medium.

Eleven operators (8 technicians, 2 radiopharmacists, 1 pharmacy resident; average years of experience in aseptic handling = 10.3 [8.7]) previously trained in both theory and practice in aseptic manipulations have been evaluated through MFT-F. All the operators carried out 3 tests over 3 successive days, over 6 weeks and 18 evaluation sessions (1 to 2 operators evaluated per week). Pre- and post-evaluation bacteriological controls of every session showed no CFU of microbiological contaminant above 5 (average 0.29 [0.63] CFU/sample for 144 plates). No nonplanar surface sample or settle plate was positive. Weekly surface sampling in the class D controlled area showed 8 positive sample, with 1 to 5 CFU/sample (average 0.73 [1.55] CFU/sample for 30 plates). These results attest the effectiveness of biodecontamination protocols. All operators validated the garbing and hygiene evaluation, with an average score of 92.7% (2.2 [1.0] deviations per operator among which, on average, 0.5 [0.5] in the prerequisites part, 1.7 [1.0] in the hygiene and garbing part, and 0 in the material handling part). All operators validated the fluorescent contamination evaluation, with an average score of 29.4 [0.8]. The smearing inside the shielded cell and on equipment did not reveal any additional fluorescent contamination. Contaminated areas were vial shields (*n* = 16; 8 operators) and disposable drape (*n* = 2; 2 operators). Mean time for a complete MFT-F was 40 [9.9] min. None of the MFT-F samples showed any visible bacterial growth after incubation. Preparation of 1 TSB-F and 1 operator evaluation was estimated at around 13.5 € (14.8 USD, see Supplementary data S4 for detail), i.e. on average 6 to 7 times less expensive than a commercial MFT kit.

## Discussion

MFT is a widely used method in hospital pharmacy to qualify both operators and, more recently, aseptic compounding robots (Krämer et al. 2016; Geersing et al., 2019; Martin et al. 2019). In radiopharmacy practice, the use of MFT has been reported for cells radiolabeling manipulations qualification (Urbano et al. 2013) but rarely enough to mimic radiopharmaceuticals preparation (Sirna et al. 2010). In parallel, the fluorescein leak test, mainly used to evaluate medical devices, also represents an interesting contribution when used to assess handling technique (Dussart et al. 2008). As the two concerns of aseptic handling and working area non-contamination are predominant in radiopharmacy practice, we proposed a combined qualification of operators through a MFT-F. To adapt to the radiation protection constrains and without commercially available alternative, we first set up a TSB-F vials preparation protocol. One of the main concerns for the preparation of TSB-F vials was fluorescein concentration to avoid quenching phenomena. In the studied concentration range, 0.01% fluorescein in TSB turned out to be the most fluorescent. At the end of the TSB-F vials production process, the pH was checked and was equal to 7.2. At this value, the predominant form of fluorescein is bi-anionic, with a high fluorescence capacity (Martin and Lindqvist, 1975; Romanchuk, 1982). Antibacterial properties of fluorescein also had to be considered. Few references in literature report the evaluation of fluorescein on bacterial strains, some of them even contradicting each other (Baab et al. 1983; Lang et al. 1972; Roy et al. 1998; Negm et al. 2016; Bouche et al. 2019). Overall, the fluorescein concentration used in our MFT-F protocol remains at least 6 folds lower than the MICs reported in literature. Moreover, in accordance with the USP and *European Pharmacopoeia* standards, a positive growth promotion test ensured TSB-F fertility. It is therefore reasonable to state that, at 0.01% concentration, fluorescein is not at risk of masking the positivity of a MFT-F. Furthermore, MFT-F controls completed with and without deliberate aseptic errors underlined the tests positivity in case of non-aseptic practices. On the other hand, these MFT-F controls highlighted the lack of sensitivity of aseptic filling tests (Kawamura and Abe, 2004). Indeed, only 7 to 9 of the 11 incubated items produced by a MFT-F control with aseptic errors showed turbidity, suggesting that minor deviances are unlikely to be detected (Kastango, 2012). To overcome this drawback, Sigward et al. suggested an intentional contamination introduction on the rubber stoppers of vials (Sigward et al. 2012). However, implementation of such a “challenged” test involving a live microorganism presents a biohazard within the pharmacy and requires dedicated facilities. Thus, we preferred to set up our MFT-F protocol as recommended both in USP and in FGMP guidelines, in routine practice facilities and under conditions closest to normal.

Operator evaluation protocol was inspired by the 2-vials preparation method of specific radiopharmaceuticals (e.g. ^99m^Tc-labelled rhenium sulfide nanocolloids, Nanocis®) (Curium, n.d.) with a larger number of transfers. The procedure of the test was design to induce enough repetition and complexity to achieve sufficient sensitivity while still mimicking a real radiopharmaceutical preparation protocol. Nevertheless, an excessively large number of units would be necessary to reach sensitivity rates recommended by ISO (International Organization for Standardization. ISO 13408-1, n.d.). In hospital pharmacy practice, this drawback can hardly be remedied due to the low number of units routinely prepared, necessarily leading to an underestimation of the microbiological contamination risk. Despite these limitations, this evaluation protocol in normal practice worst-case conditions is suitable for small to medium-sized radiopharmacy departments, with relatively few staff to qualify.

All operators passed the garbing and hygiene evaluation part, however, the relatively large number of deviations in the hygiene and gowning section (mainly concerning radiation protection elements wearing, sufficient hand washing and correct steps order) resulted in renewed advices to operators on good gowning and hygiene practices. Test duration was highly variable depending on the operators but did not seem to be correlated with experience in aseptic handling. All operators validated the fluorescent contamination part of the test, although 1 to 2 contaminations were reported in at least 1 MFT-F for 8 operators. The most frequently reported contamination was on rubber stoppers of vials, also regularly encountered in routine radiopharmacy practice. Interestingly, we noticed that nearly 20% of patient syringes (*n* = 35 among 198 syringes) were contaminated with a few drops of TSB-F inside their cap. Only 1 operator produced, at the end of the 3 MFT-F performed, 18 patient syringes without cap contamination. This error, not listed in the operator evaluation form, is nevertheless a preventable factor of radiation exposure for both the patient and the staff administering the radiopharmaceutical. More significantly, such a discrepancy in daily practice can distort the activity actually injected to the patient. All operators have therefore been made aware of this fact and were asked to remain vigilant about the non-contamination of patient syringes cap.

Implementing a MFT-F for operators qualification in our radiopharmacy unit completed the existing sterile compounding instruction. After this initial 3-tests evaluation, annual periodic requalification of operators should be planned to promptly identify and correct any deviations in good sterile compounding practices. Within the framework of our sterile compounding training uptade, we could consider completing or replacing current theoretical training by original and didactic online learning modules. Moreover, evaluation of the acquired knowledge through a written examination would fulfill USP chapter 797 requirements.

## Conclusions

Ensuring the quality of professional practices in hospital activities such as radiopharmacy is a mission of the hospital pharmacist, helping to guarantee reliability, relevance and validity of diagnostic and therapeutic approaches. Combined fluorescein/media fill test is a qualification method as part of the quality management system to control biological and environmental contamination risks. It may also be useful to highlight deviations from good radiopharmacy practices. Combined MFT-F, embedded in a comprehensive sterile compounding training program, appears as a promising tool to increase both the sterile compounding safety and awareness of radioactive contamination in radiopharmacy.

## Supplementary information

**Additional file 1.** S1: TSB-F preparation protocol; S2: Operator evaluation form; S3: Detailed MFT-F protocol; S4: MFT-F cost table.

## Data Availability

Supplementary Materials are available online. S1: TSB-F preparation protocol; S2: Operator evaluation form; S3: Detailed MFT-F protocol; S4: MFT-F cost table.

## References

[CR1] Amalric P (1972). Fluorescein angiography: international symposium, Albi, 1969: Proceedings S Karger AG.

[CR2] Baab DA, Broadwell AH, Williams BL (1983). A comparison of antimicrobial activity of four disclosant dyes. J Dent Res.

[CR3] Biechlin M-L, Léger S, Vial F, Desruet M-D (2007). How to combine hygiene and radiation protection for radiopharmaceuticals preparation? Analysis in France and propositions. Nucl Med Commun.

[CR4] Bouche T, Auzou M, Daurel C, Quintyn J, Join-Lambert O, Guérin F (2019). In vitro evaluation of the antibacterial activity of fluorescein® 0.5% eye drops. Acta Ophthalmol.

[CR5] Council of Europe, 2018 (2018). European Pharmacopoeia 9th Ed. Main Volume 9.6 2.6.1: Sterility.

[CR6] Covens P, Berus D, Caveliers V, Struelens L, Verellen D (2012). Skin contamination of nuclear medicine technologists: incidence, routes, dosimetry and decontamination. Nucl Med Commun.

[CR7] Curium (n.d.), NANOCIS summary of product characteristics (updated 01/2018) [Online] https://www.curiumpharma.com/wp-content/uploads/2018/05/T1700nJ.pdf, accessed 2020/03/06.

[CR8] Decristoforo C, Patt M (2017). Are we “preparing” radiopharmaceuticals?. EJNMMI Radiopharm Chem.

[CR9] Decristoforo C, Penuelas I, Elsinga P, Ballinger J, Winhorst AD, Verbruggen A (2014). Radiopharmaceuticals are special, but is this recognized? The possible impact of the new clinical trials regulation on the preparation of radiopharmaceuticals. Eur J Nucl Med Mol Imaging.

[CR10] Dussart C, Favier B, Gilles L, Camal I, Almeras D, Latour JF, Grelaud G (2008). Continuous training program for technicians handling antineoplastic drugs and occupational exposure risk. Bull Cancer (Paris).

[CR11] Elsinga P, Todde S, Penuelas I, Meyer G, Farstad B, Faivre-Chauvet A (2010). Guidance on current good radiopharmacy practice (cGRPP) for the small-scale preparation of radiopharmaceuticals. Eur J Nucl Med Mol Imaging.

[CR12] EudraLex (2015). Volume 4 - Good Manufacturing Practice (GMP) guidelines - European Commission.

[CR13] Favier B, Labrosse H, Gilles-Afchain L, Cropet C, Perol D, Chaumard N (2012). The PhaSeal® system: impact of its use on workplace contamination and duration of chemotherapy preparation. J Oncol Pharm Pract.

[CR14] Garrigue P, Montana M, Ventre C, Savry A, Gauthier-Villano L, Pisano P, Pourroy B (2016). Safe cytotoxic drug preparation using closed-system transfer device: technical and practical evaluation of a new device (Vialshield/Texium) comparatively to a reference one (Phaseal). Int J Pharm Compd.

[CR15] Geersing TH, Franssen EJF, Pilesi F, Crul M (2019). Microbiological performance of a robotic system for aseptic compounding of cytostatic drugs. Eur J Pharm Sci.

[CR16] Heller SL (1996). Radiation safety in the central radiopharmacy. Semin Nucl Med.

[CR17] International Organization for Standardization. ISO 13408-1 aseptic processing of health care products. Part 1: general requirements. https://www.iso.org/obp/ui/#iso:std:iso:13408:-1:ed-2:v1:en, accessed 2020/03/06 (n.d.).

[CR18] Kastango ES (2012). Challenging our aseptic skills using more-rigorous media-fill tests. Am J Health Syst Pharm.

[CR19] Kawamura K (2002). The media fill (simulation) test is the best method to evaluate aseptic processing. PDA J Pharm Sci Technol.

[CR20] Kawamura K, Abe H (2004). A novel approach to the statistical evaluation of media fill tests by the difference from no contamination data. PDA J Pharm Sci Technol.

[CR21] Krämer I, Federici M, Kaiser V, Thiesen J (2016). Media-fill simulation tests in manual and robotic aseptic preparation of injection solutions in syringes. J Oncol Pharm Pract.

[CR22] Lang NP, Ostergaard E, Loe H (1972). A fluorescent plaque disclosing agent. J Periodontal Res.

[CR23] Martin MM, Lindqvist L (1975). The pH dependence of fluorescein fluorescence. J Lumin.

[CR24] Martin T, Moyon A, Fersing C, Terrier E, Gouillet A, Giraud F (2019). Have you looked for “stranger things” in your automated PET dose dispensing system? A process and operators qualification scheme. EJNMMI Radiopharm Chem.

[CR25] Mullarkey T (2009). Pharmacy compounding of high-risk level products and patient safety. Am J Health Syst Pharm.

[CR26] Myers CE (2013). History of sterile compounding in U.S. hospitals: learning from the tragic lessons of the past. Am J Health Syst Pharm.

[CR27] Negm NA, Abou Kana MTH, Abd-Elaal AA, Elwahy AHM (2016). Fluorescein dye derivatives and their nanohybrids: synthesis, characterization and antimicrobial activity. J Photochem Photobiol B.

[CR28] Nemec EC, Petraglia C, Mattison MJ (2016). Design considerations of a compounded sterile preparations course. Am J Pharm Educ.

[CR29] Romanchuk KG (1982). Fluorescein. Physicochemical factors affecting its fluorescence. Surv Ophthalmol.

[CR30] Roy JJ, Lau A, McFee DG (1998). Antibacterial activity of fluorescein. CMAJ Can Med Assoc J J Assoc Medicale Can.

[CR31] Segner V, Kimbel R, Jochems P, Heinemann A, Letzel S, Wollschläger D, Rossbach B (2017). Liquid release as a source of potential drug exposure during the handling of intravenous infusions in nursing. Int Arch Occup Environ Health.

[CR32] Shehab N, Brown MN, Kallen AJ, Perz JF (2018). U.S. compounding pharmacy-related outbreaks, 2001–2013: public health and patient safety lessons learned. J Patient Saf.

[CR33] Sigward E, Fourgeaud M, Vazquez R, Guerrault-Moro M-N, Brossard D, Crauste-Manciet S (2012). Aseptic simulation test challenged with microorganisms for validation of pharmacy operators. Am J Health Syst Pharm.

[CR34] Sirna V, Garaboldi L, Papi S, Martano L, Omodeo Salè E, Paganelli G, Chinol M (2010). Testing of microbial contamination during the preparation of the radiocompound [^90^Y] DOTATOC for clinical trials: a process validation study by media fill approach. Q J Nucl Med Mol Imaging.

[CR35] Staes C, Jacobs J, Mayer J, Allen J (2013). Description of outbreaks of health-care-associated infections related to compounding pharmacies, 2000–12. Am J Health Syst Pharm.

[CR36] Tazrart A, Bérard P, Leiterer A, Ménétrier F (2013). Decontamination of radionuclides from skin: an overview. Health Phys.

[CR37] Urbano N, Modoni S, Schillaci O (2013). Media fill test for validation of autologous leukocytes separation and labelling by ^99m^Tc-HmPAO. Nucl Med Biol.

[CR38] USP<71> (2018). Sterility tests. The United States Pharmacopeia and National Formulary. USP 41-NF 36 U.S.

[CR39] USP<797> (2017). Pharmaceutical compounding—sterile preparations. The United States Pharmacopeia, 40th rev., and the National Formulary. 35 ed.

