# Supplementary material for: Implementation and validation of an in-house combined fluorescein/media-fill test to qualify radiopharmacy operators
Source: EJNMMI Radiopharm Chem. 2021 Jan 7;6:2. doi: 10.1186/s41181-020-00117-6 (PMC7790972; doi:10.1186/s41181-020-00117-6)
Supplement: Supplementary file 1 — Additional file 1. S1: TSB-F preparation protocol; S2: Operator evaluation form; S3: Detailed MFT-F protocol; S4: MFT-F cost table. [file 41181_2020_117_MOESM1_ESM.zip › Suplementary data S1.docx]

**TSB-F preparation protocol**

This step was carried out inside a class II laminar airflow hood (BH-EN-2003 D, Faster S.R.L., Ferrara, Italy) properly disinfected and placed in a class D controlled area. Vial preparation procedure is shown in the following figure.


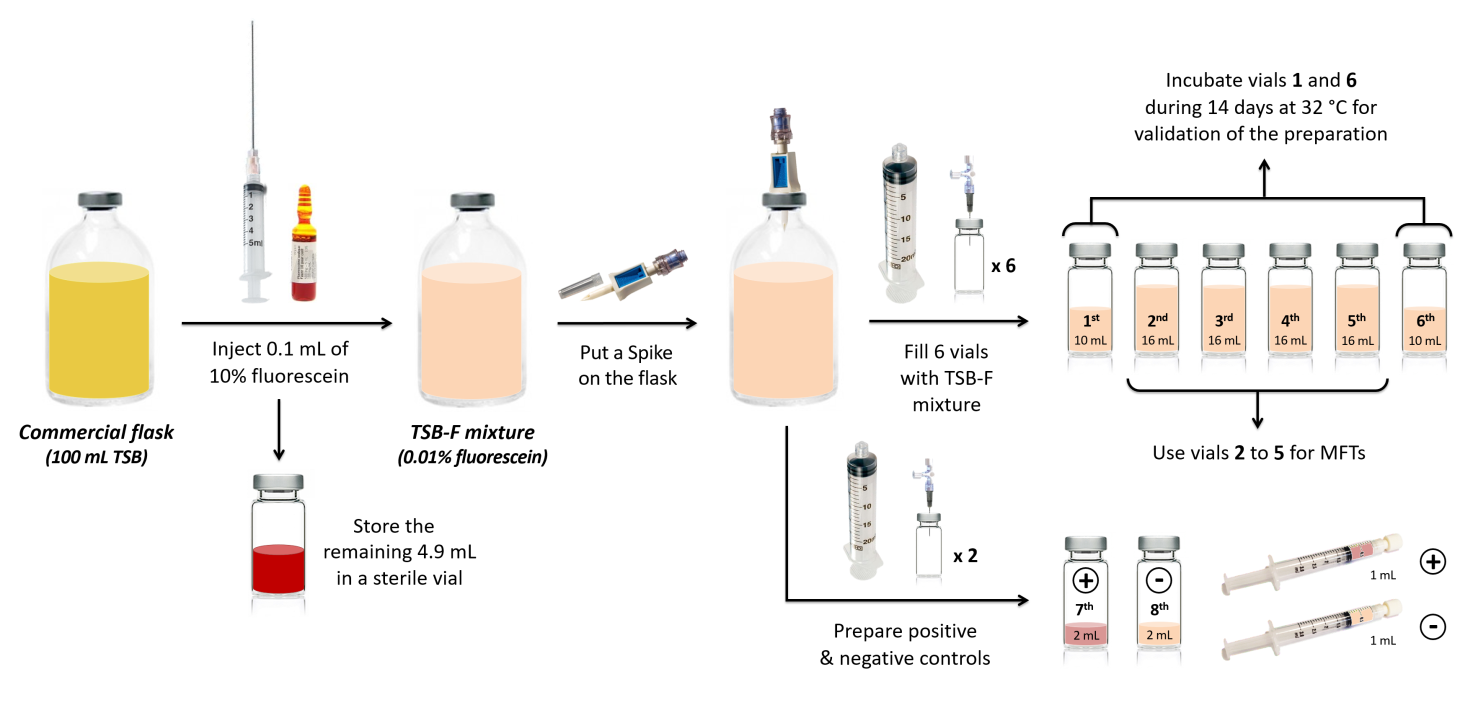


**Step-by-step instructions**:

1. Identify 8 sterile 15 mL elution vials (TC-ELU-5®, CIS bio international, Saclay, France) from number 1 to number 8.
2. Insert into each vial a 22G 30 mm needle (BD Microlance™ 3, Becton Dickinson S.A., Franklin Lakes, NJ, USA) with a 2-way valve (Discofix®, B. Braun, Melsungen, Germany).
3. To a 100 mL sealed sterile tryptic soy broth flask (VWR, Radnor, PA), add aseptically 100 µL of fluorescein 10% solution (Fluorescéine sodique Faure 10 pour cent®, solution for injection, 5 mL glass vial, SERB, Brussels, Belgium) using a 1 mL syringe (BD Plastipak™, Becton Dickinson S.A., Franklin Lakes, NJ, USA) with a 23G 60 mm needle (Sterican®, B. Braun, Melsungen, Germany).
4. Insert into the TSB flask a transfer spike (CODAN Spike, CODAN, Lensahn, Germany).
5. Withdraw 10 mL of TSB-F mixture with a 20 mL syringe (BD Plastipak™ Luer-Lok™, Becton Dickinson S.A., Franklin Lakes, NJ, USA) to fill elution vial 1.
6. Fill vials 2 to 5 with 16 mL TSB-F each, using the same 20 mL syringe.
7. Fill vial 6 with 10 mL TSB-F using the same 20 mL syringe.
8. Fill vials 7 and 8 with 2 mL TSB-F each to serve as positive and negative control, respectively.
9. Fill two 3 mL syringes (BD Luer-Lok™, Becton Dickinson S.A., Franklin Lakes, NJ, USA) with 1 mL TSB-F each.
10. Cap the 2 syringes with a luer-lock stopping plug (COMBI-LOCK, CODAN, Lensahn, Germany) to serve as positive and negative control, respectively.
11. Spoil positive control vials with a few microliters of contaminated TSB medium.
12. Incubate vial 1 and 6 during 14 days at 32 °C for aseptic validation of the manipulation.
13. Store vials 2 to 5 at 4-8 °C before use. Vials should be prepared the same week as the MFT is performed.
