# Supplementary material for: Implementation and validation of an in-house combined fluorescein/media-fill test to qualify radiopharmacy operators
Source: EJNMMI Radiopharm Chem. 2021 Jan 7;6:2. doi: 10.1186/s41181-020-00117-6 (PMC7790972; doi:10.1186/s41181-020-00117-6)
Supplement: Supplementary file 1 — Additional file 1. S1: TSB-F preparation protocol; S2: Operator evaluation form; S3: Detailed MFT-F protocol; S4: MFT-F cost table. [file 41181_2020_117_MOESM1_ESM.zip › Suplementary data S2.docx]

Date of evaluation: ______________________________

Name/position of evaluator: ______________________________

Name/position of the operator: ______________________________

Years of experience in aseptic handling: ____________

Type of evaluation: ❑ Initial assessment (MFT number ❑ 1 ❑ 2 ❑ 3)

❑ Periodic qualification

❑ Other (specify): ____________________________

**Part I - Garbing and hygiene evaluation (a negative answer being considered as an error)**

| ***Entry into the radiopharmaceutical preparation laboratory: prerequisites*** | | Yes / No | | Commentaries |
| --- | --- | --- | --- | --- |
| 1 | Wear a passive dosimeter on the chest |  |  |  |
| 2 | Wear an operational dosimeter on the chest |  |  |  |
| 3 | Wear a thermoluminescent dosimetric ring |  |  |  |
| 4 | Wear a clean outfit specific for the radiopharmacy unit |  |  |  |
| 5 | Short neat natural nails, with no nail polish or artificial nails |  |  |  |
| 6 | Wear no jewelry, watches, wedding rings… |  |  |  |
| 7 | Wear no makeup |  |  |  |
| 8 | Long hair pulled back or up in a bun, if applicable |  |  |  |
| 9 | Beard/facial hair neat and trimmed |  |  |  |
| 10 | No visible piercings |  |  |  |
| 11 | Wear closed-toe shoes specific to the radiopharmacy unit |  |  |  |
| 12 | No introduction of food, chewing gum, water or personal objects |  |  |  |
| 13 | No rashes, weeping sores, sunburn, conjunctivitis or respiratory infection |  |  |  |
| ***Hygiene and gowning*** | | Yes / No | | Commentaries |
| 14 | Don a shielded apron |  |  |  |
| 15 | Put on overshoes properly and move in the clean zone of the anteroom |  |  |  |
| 16 | Respect the "dirty to clean" zones delimitation in the anteroom |  |  |  |
| 17 | Put on head cover properly, with no hair sticking out |  |  |  |
| 18 | Don and tie mask properly (blue side out) ± beard cover if applicable |  |  |  |
| 19 | Wash hands & forearms with soap for at least 30 sec |  |  |  |
| 20 | Don gown properly |  |  |  |
| 21 | Wash hands with alcohol-based handrub gel |  |  |  |
| 22 | Disinfection of the thermoluminescent dosimetric ring |  |  |  |
| 23 | Put on non-sterile gloves |  |  |  |
| 24 | Sequence of steps 14 to 22 observed |  |  |  |
| ***Material handling*** | | Yes / No | | Commentaries |
| 25 | Disinfection of the package of the kit contents (70% isopropyl alcohol) |  |  |  |
| 26 | Cleaning of the non-disposable equipment (detergent-disinfectant solution) |  |  |  |
| 27 | Disinfection of the septum of each vial (alcoholic 2% chlorhexidine) |  |  |  |
| 28 | Correct installation of the sterile disposable drape inside the glovebox |  |  |  |
| 29 | Disposable equipment packaging correctly peeled |  |  |  |
| 30 | No aseptic error during the MFT |  |  |  |

Validation criteria: Total score > 90% (≤ 3 errors) Number of errors: ___________

**Result: Garbing and hygiene evaluation ❑ Passed**

**❑ Not passed**

**Part II – Fluorescent contamination evaluation**

Specific contaminated areas or equipment (marked with a cross):

| ***Area or material*** | ***Number of points if fluorescence*** | ***Fluorescence detection*** | | | ***Precise location of the contamination*** |
| --- | --- | --- | --- | --- | --- |
|  |  | **None** | **Direct** | **Smearing** |  |
| Gloves | - 2 | ❑ | ❑ | ❑ | 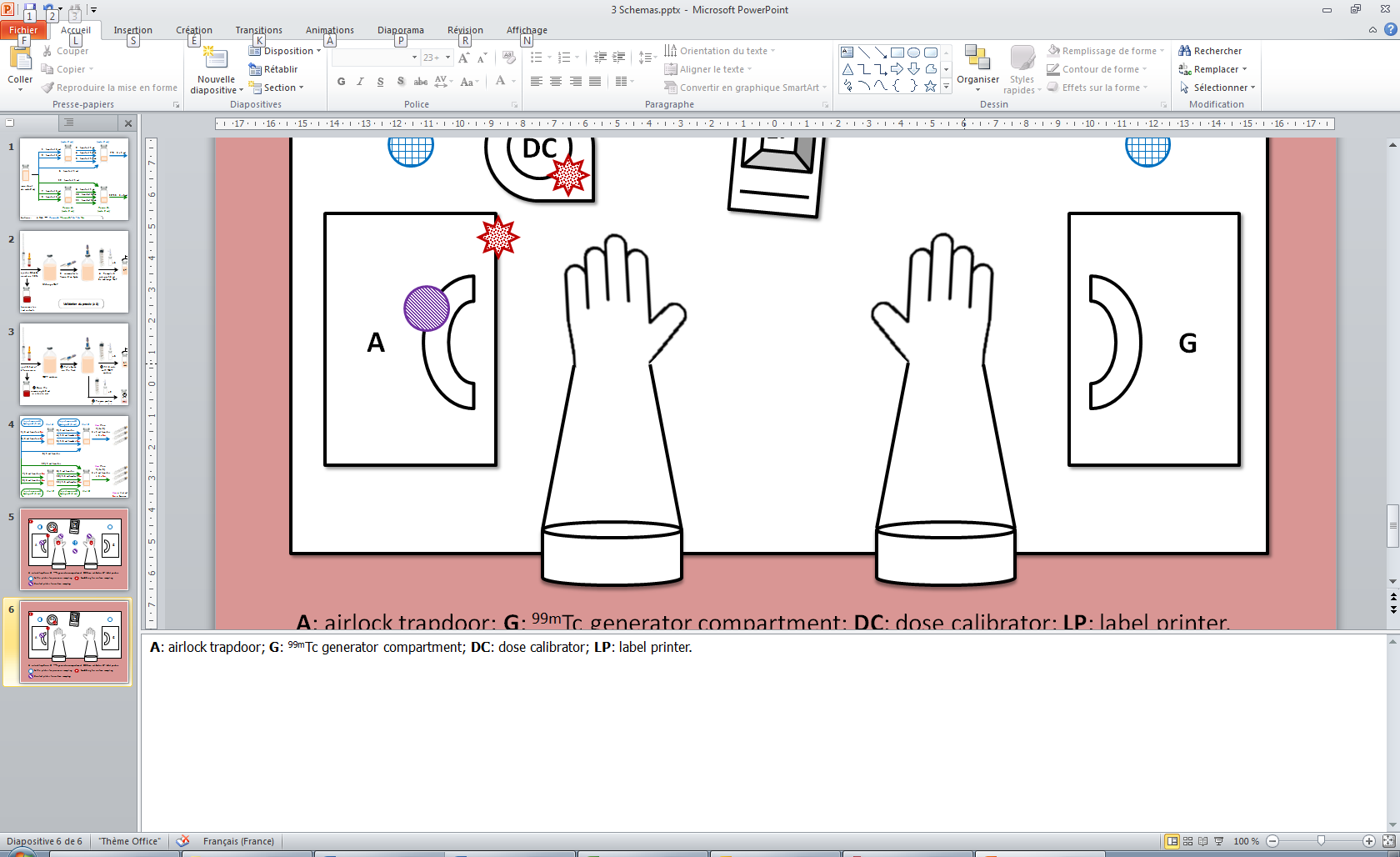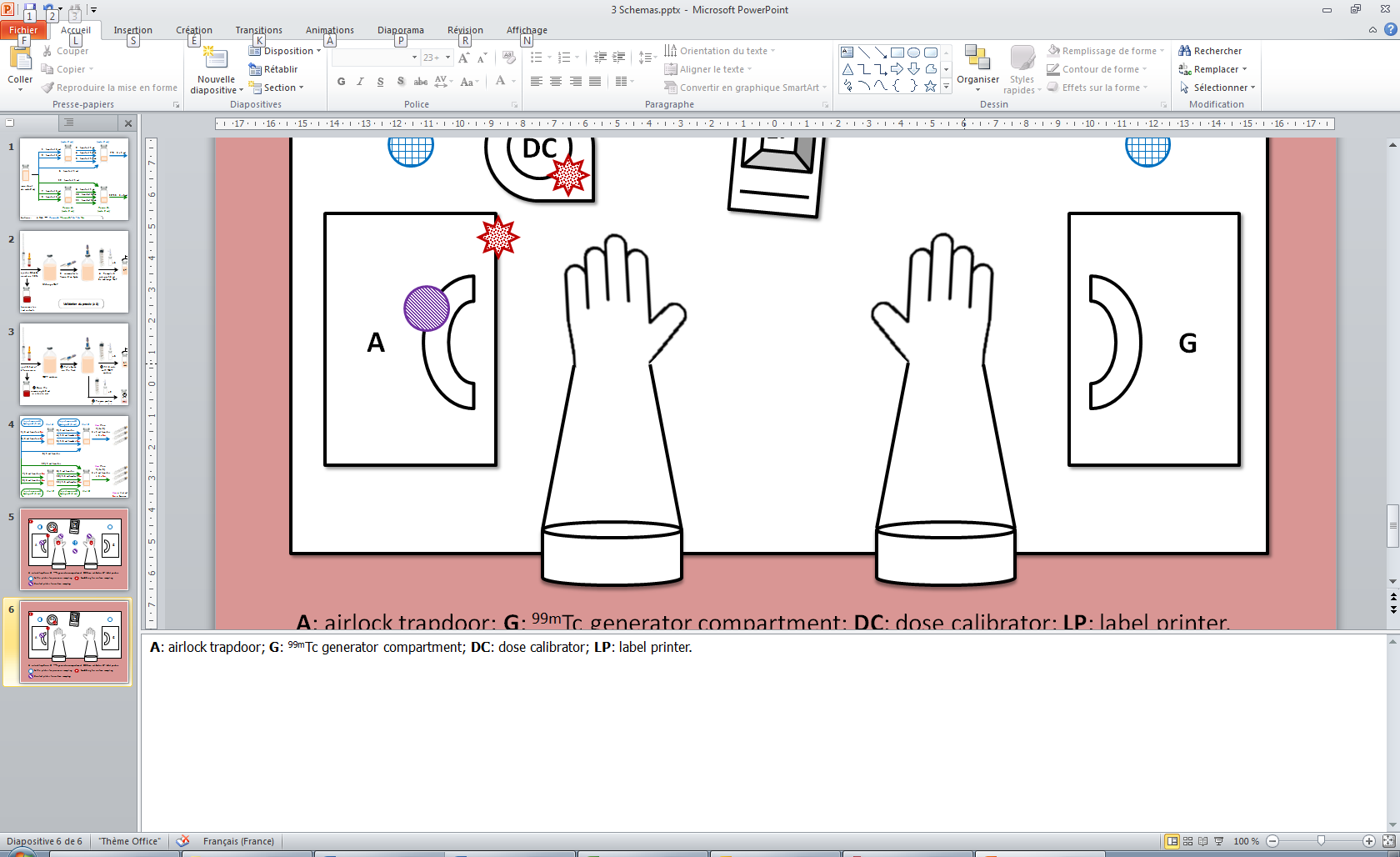 |
| Disposable drape | - 1 | ❑ | ❑ | ❑ | 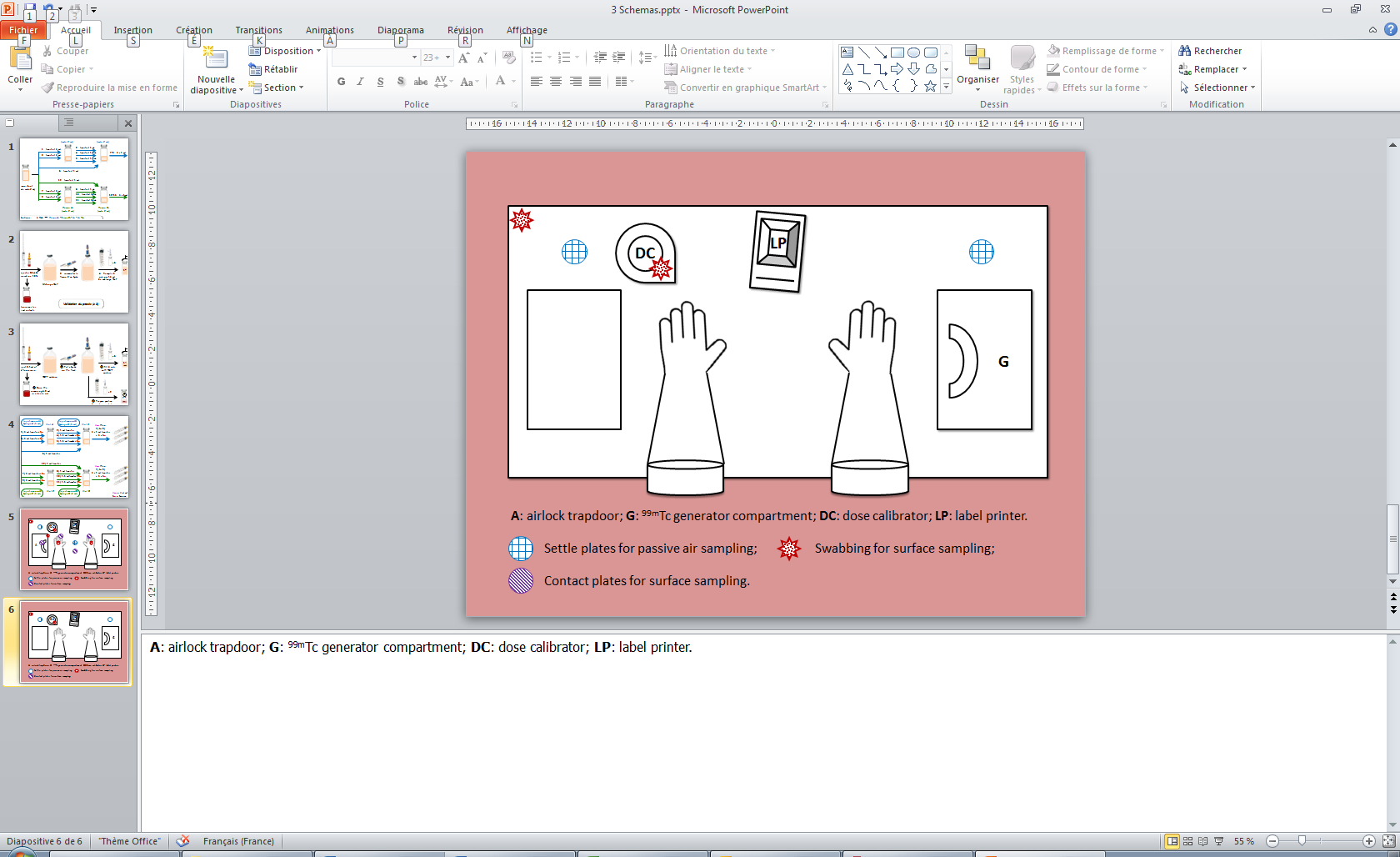 |
| Working area | - 2 | ❑ | ❑ | ❑ | 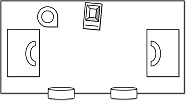 |
| Bottom of the dipper | Eliminatory | ❑ | ❑ | ❑ | 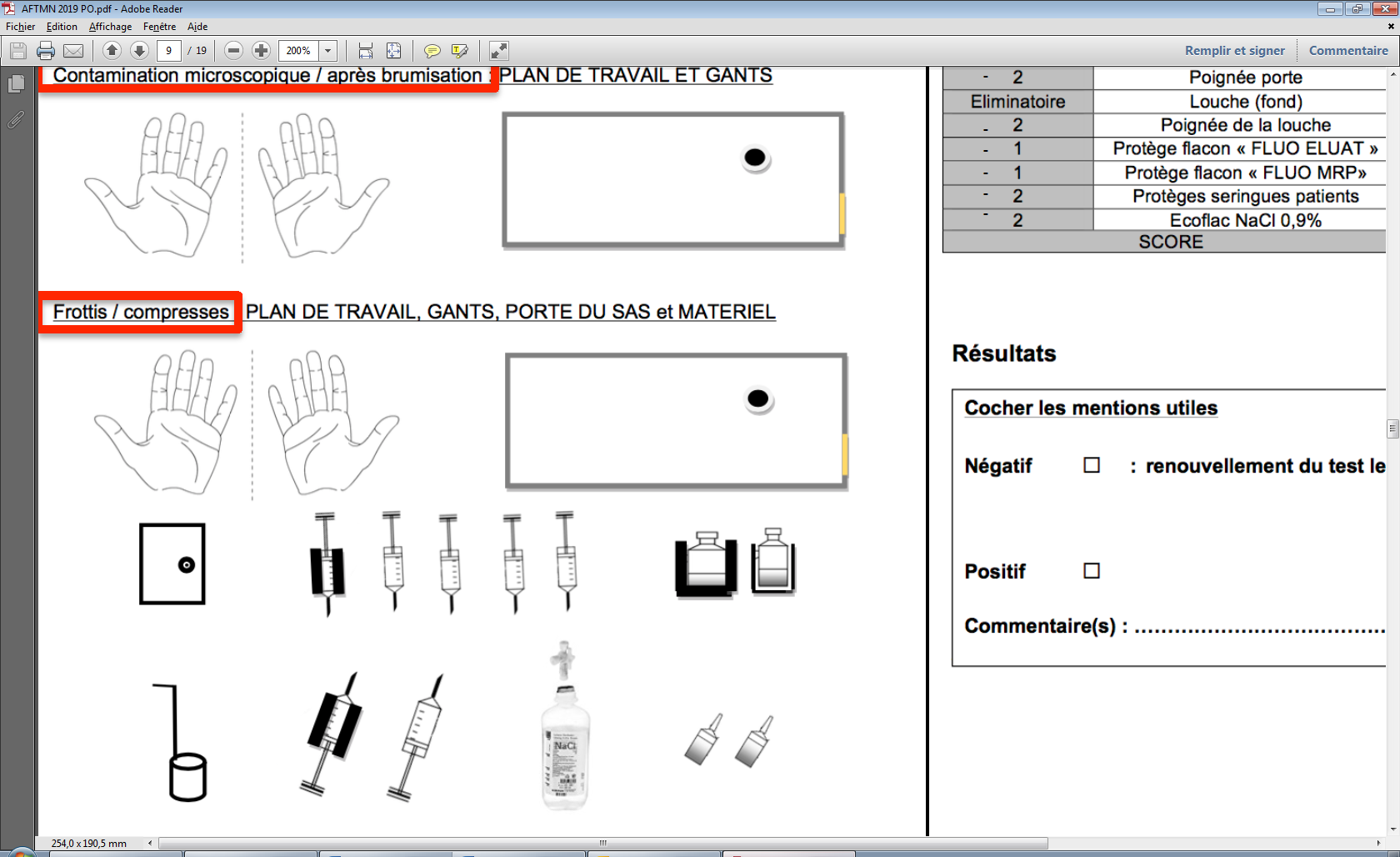 |
| “Eluate” vial shield | - 1 | ❑ | ❑ | ❑ | 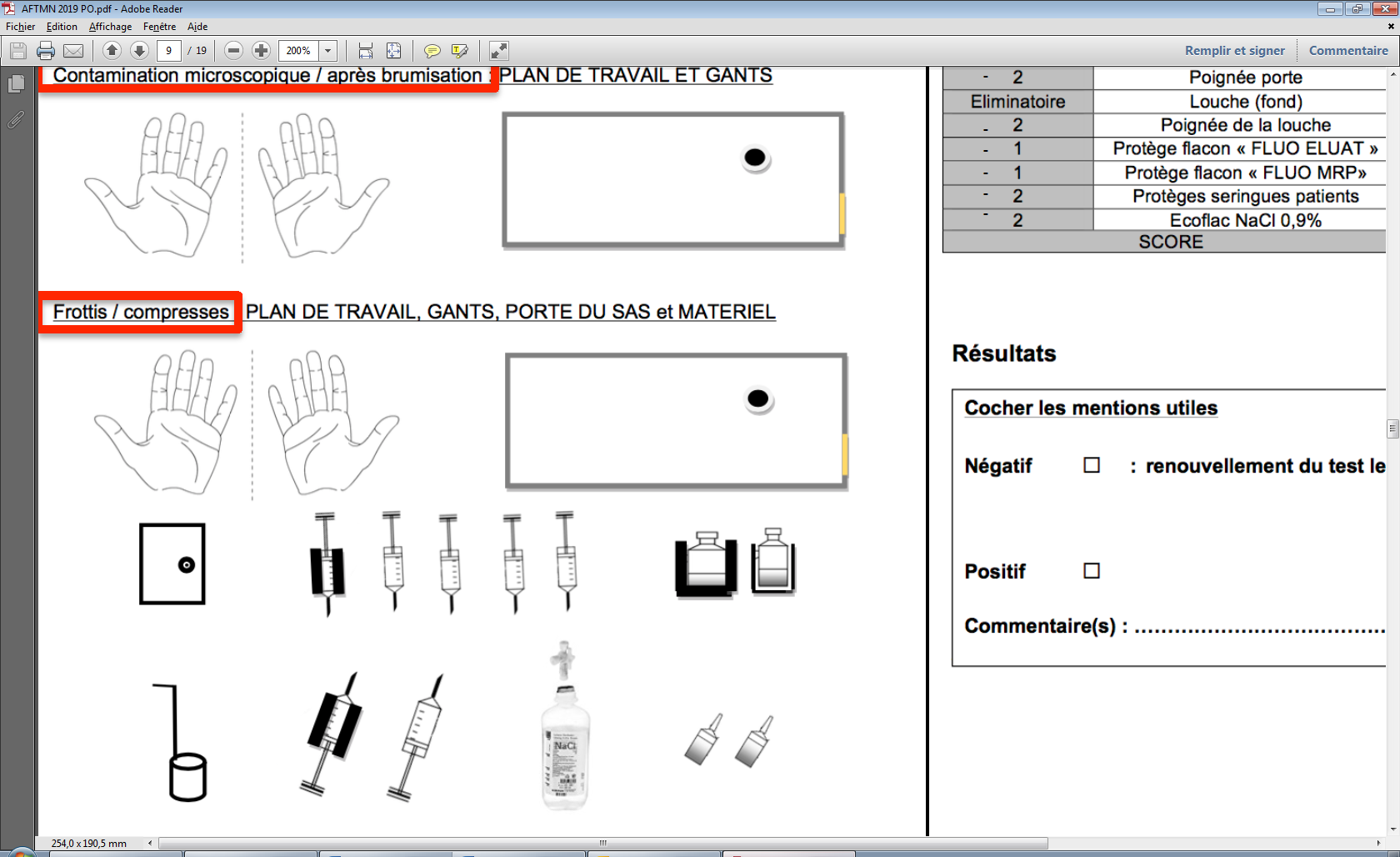 |
| Vial A shield | - 1 | ❑ | ❑ | ❑ | 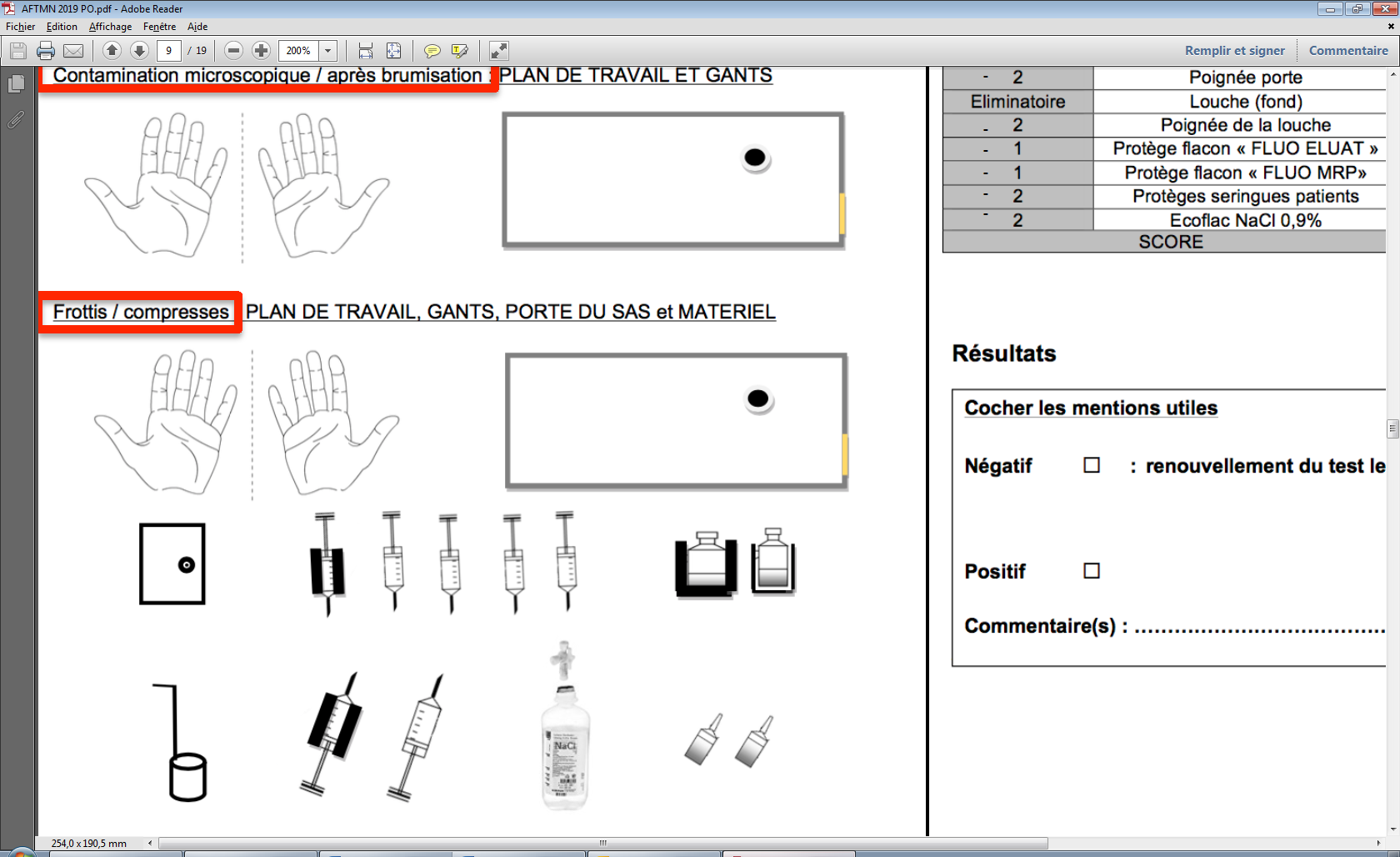 |
| Vial B shield | - 1 | ❑ | ❑ | ❑ | 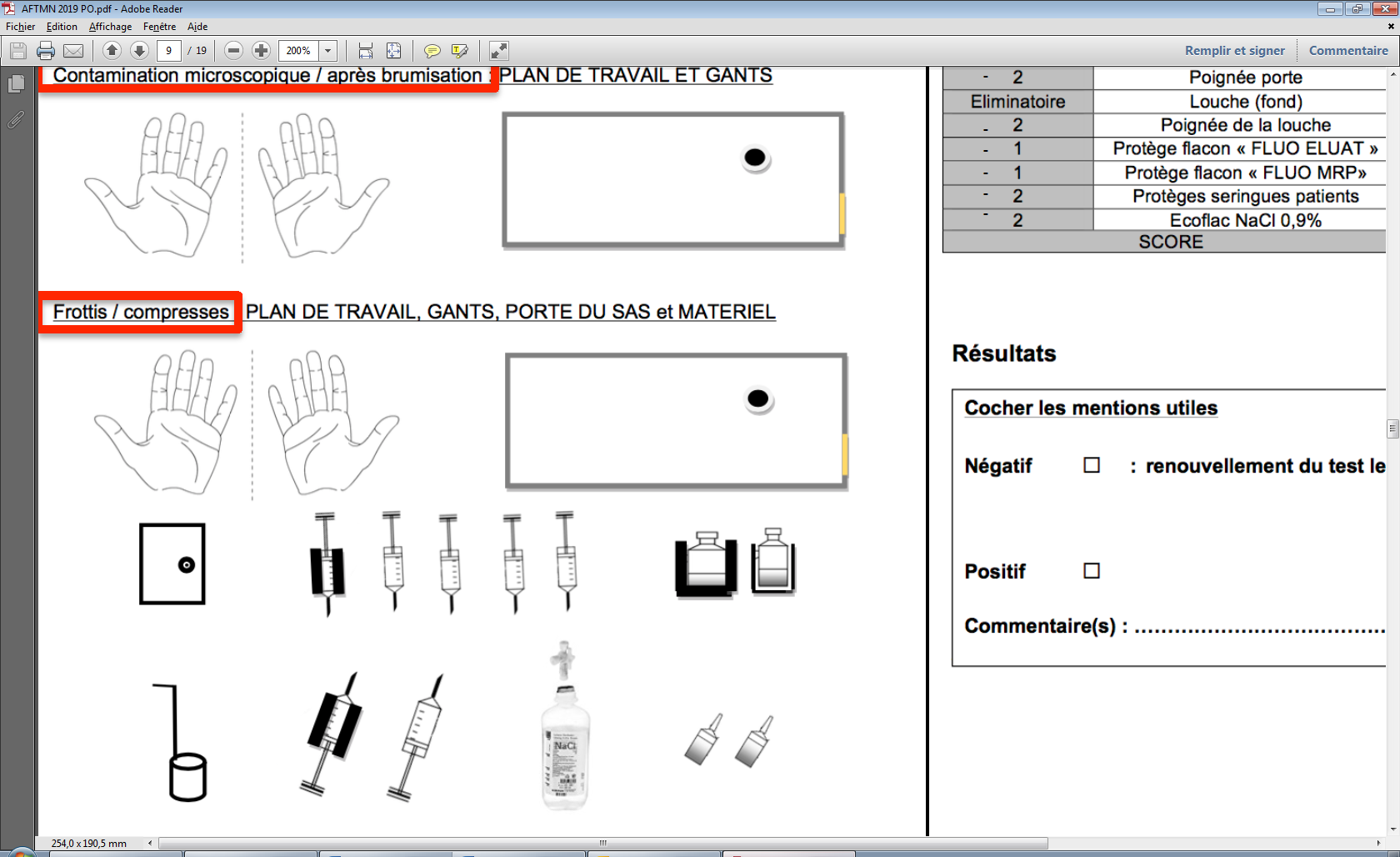 |
| Vial C shield | - 1 | ❑ | ❑ | ❑ | 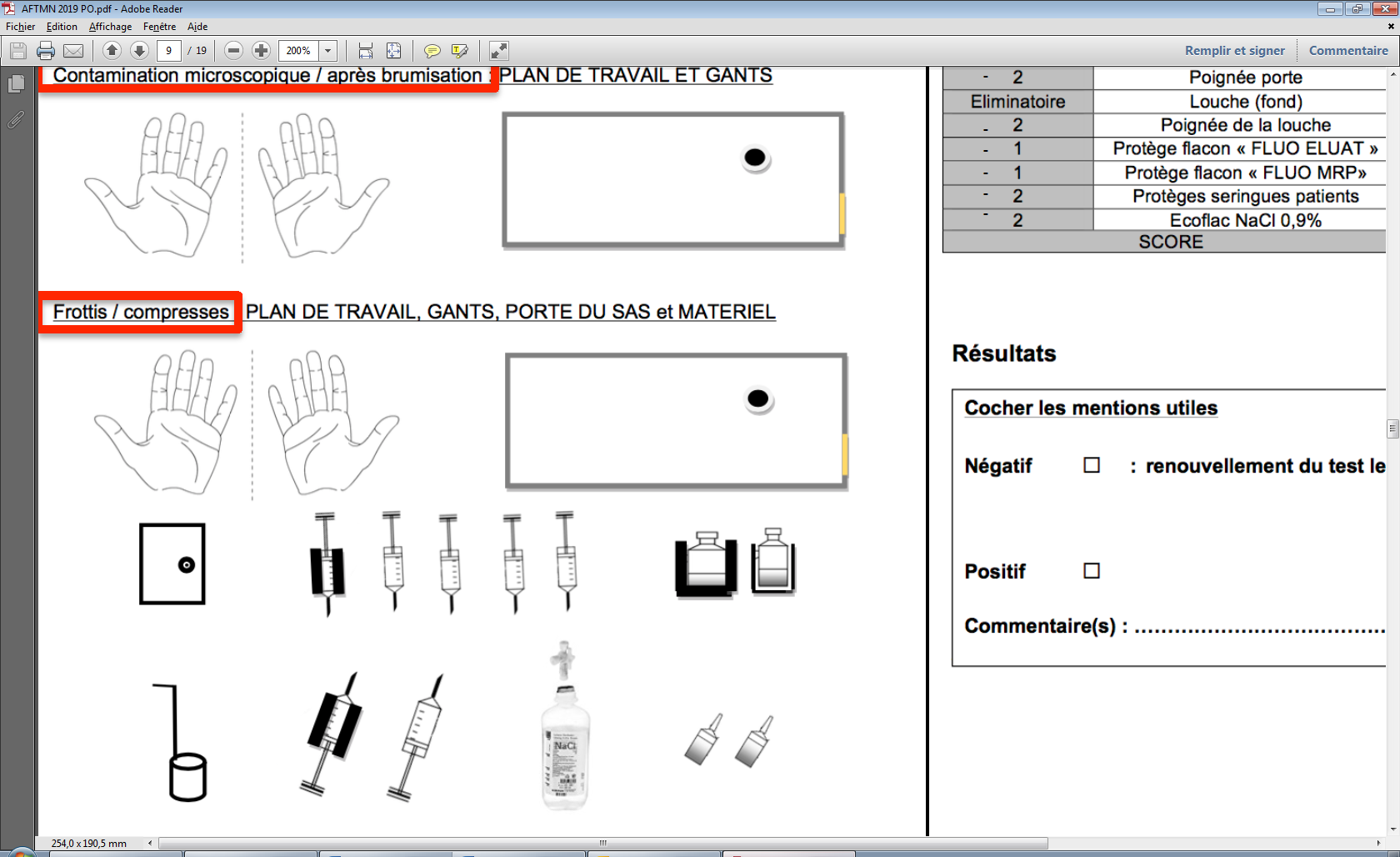 |
| Vial D shield | - 1 | ❑ | ❑ | ❑ | 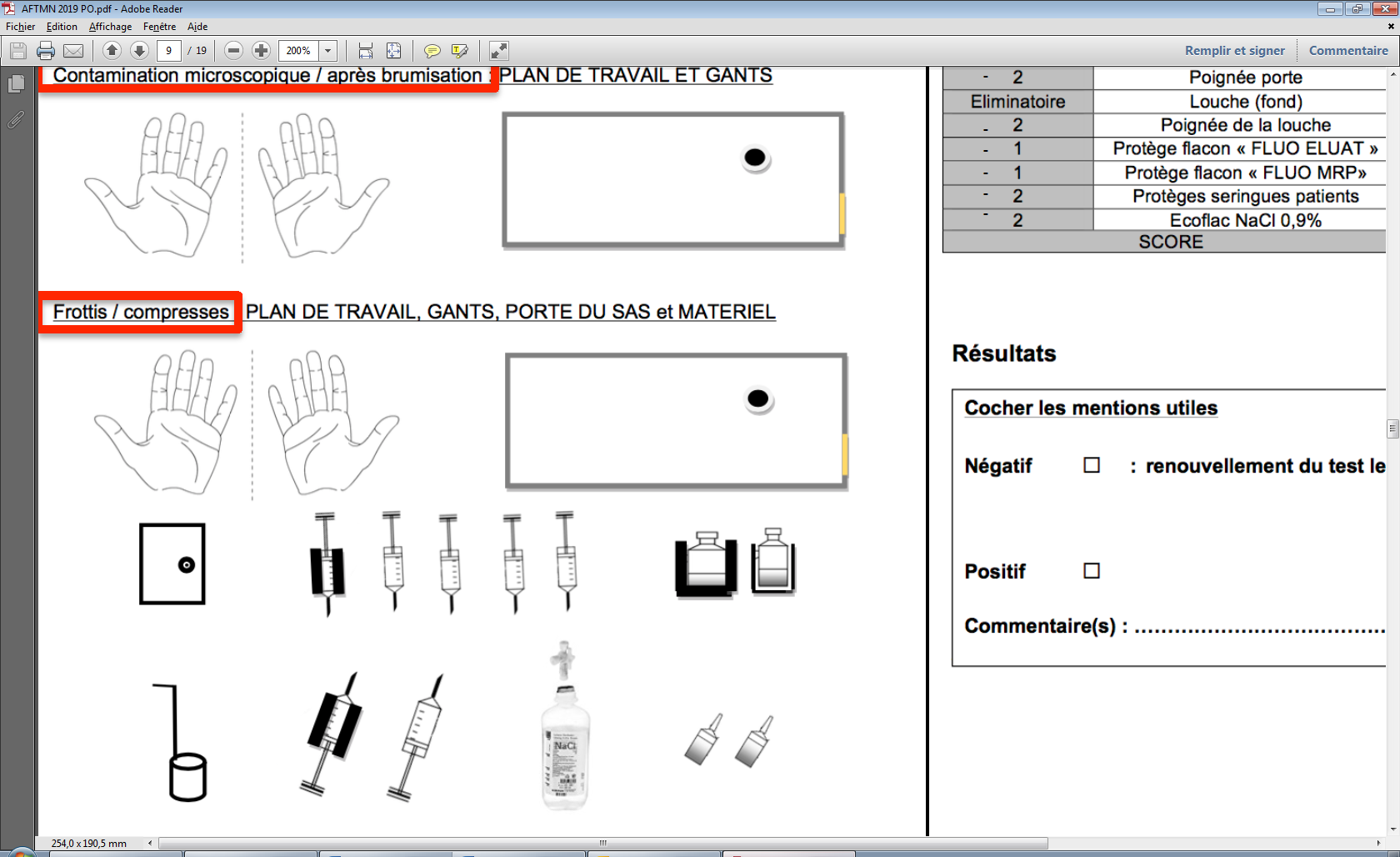 |
| Syringe A shield | - 2 | ❑ | ❑ | ❑ | 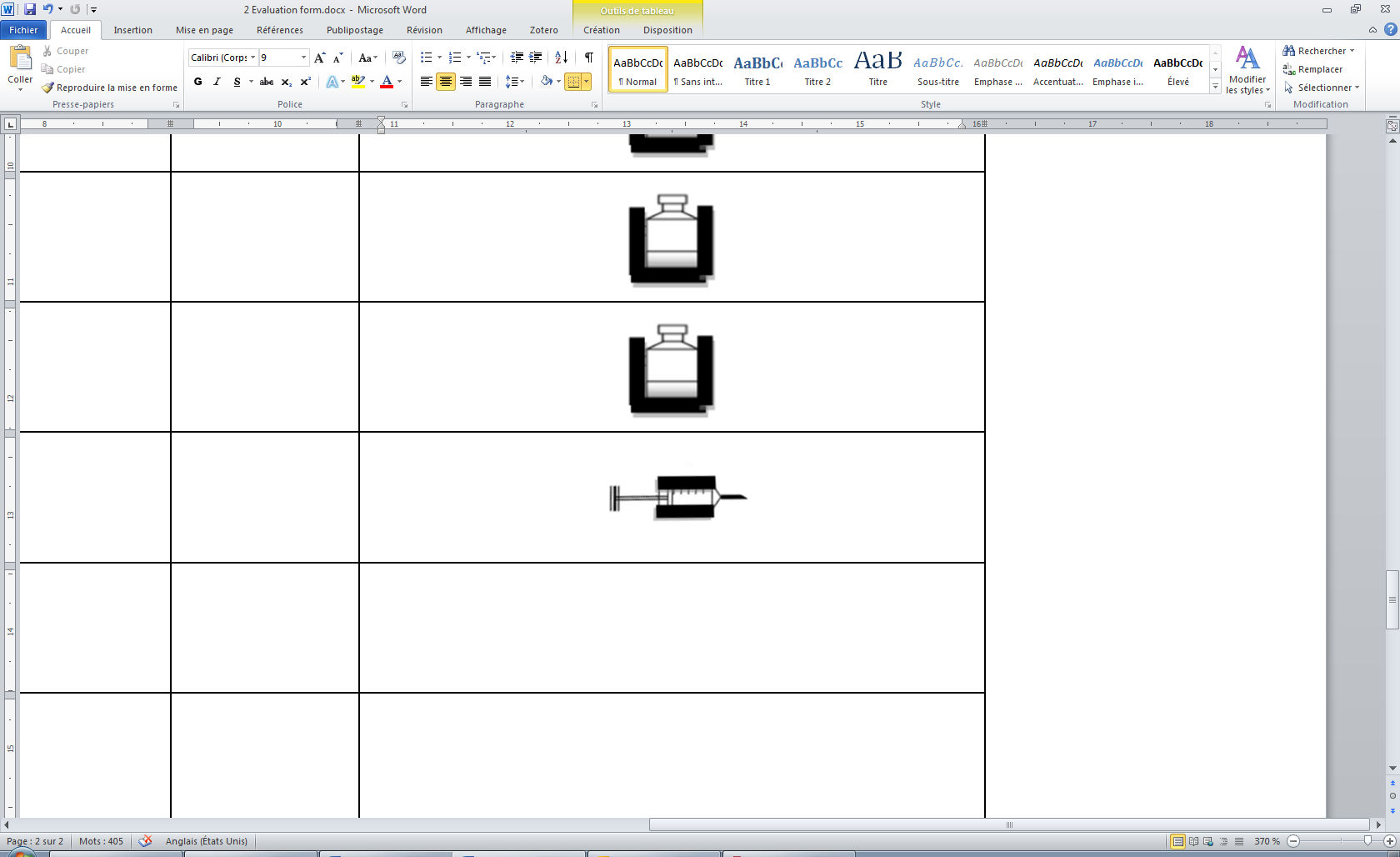 |
| Syringe B shield | - 2 | ❑ | ❑ | ❑ | 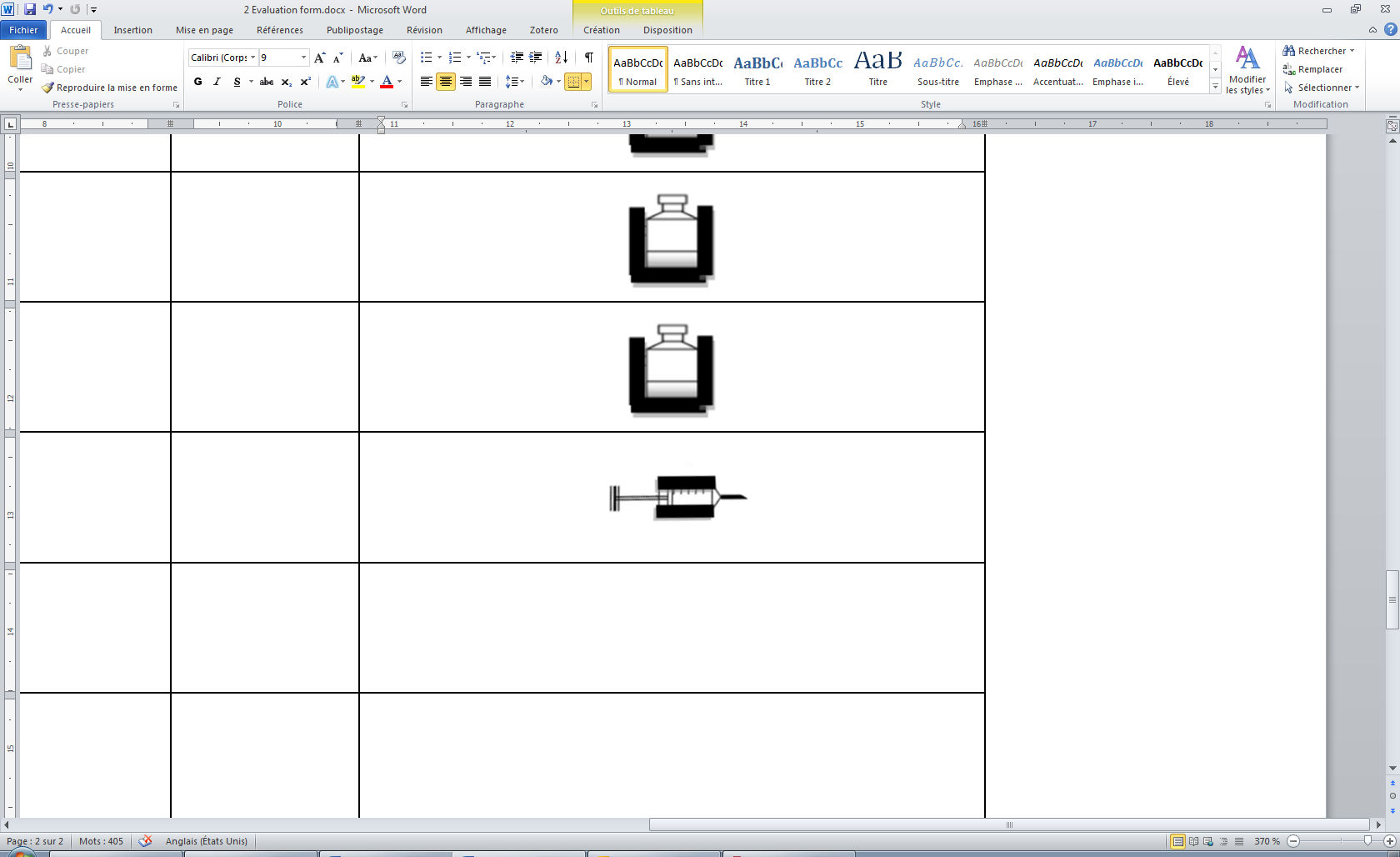 |
| Syringe C shield | - 2 | ❑ | ❑ | ❑ | 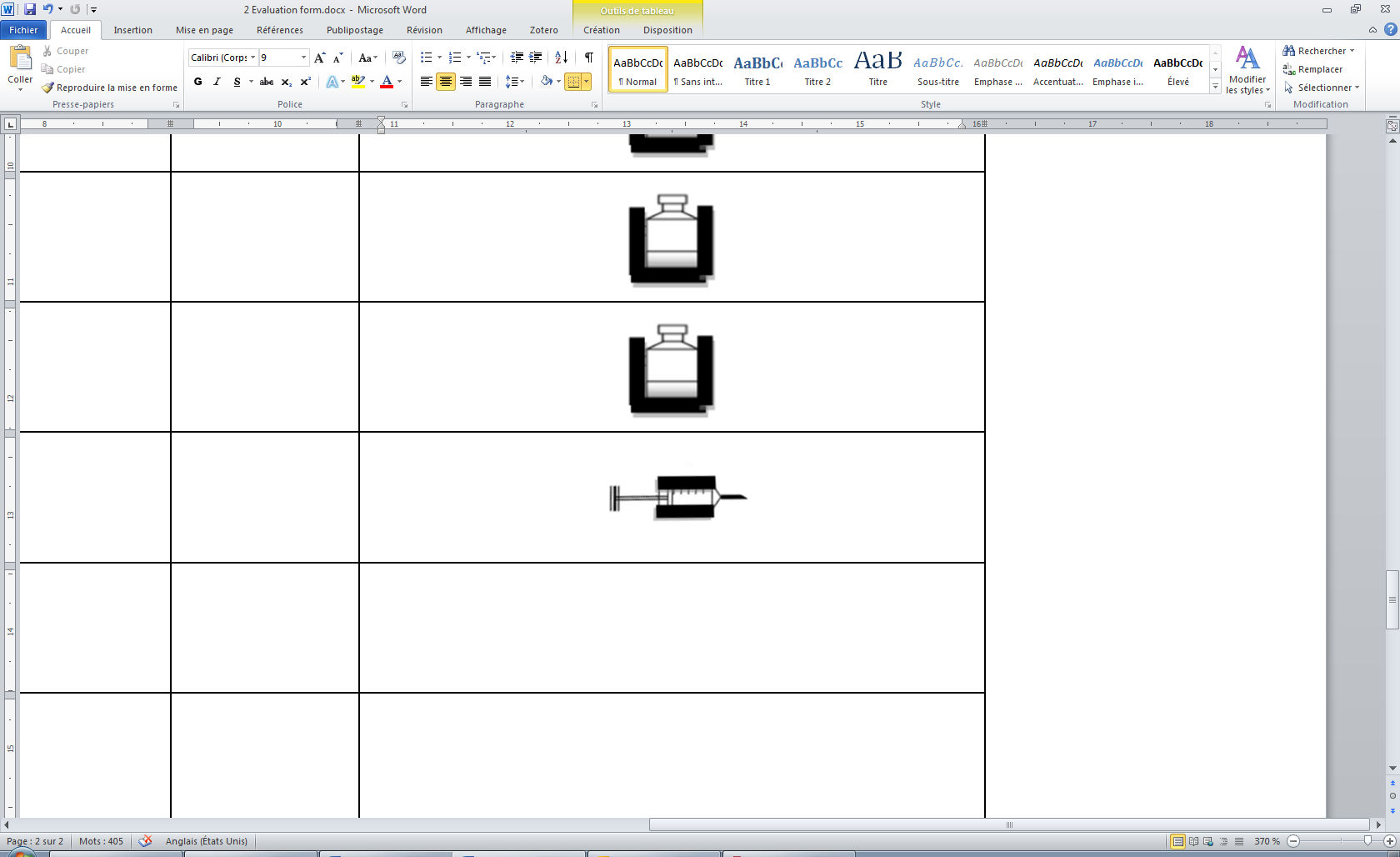 |
| Syringe D shield | - 2 | ❑ | ❑ | ❑ | 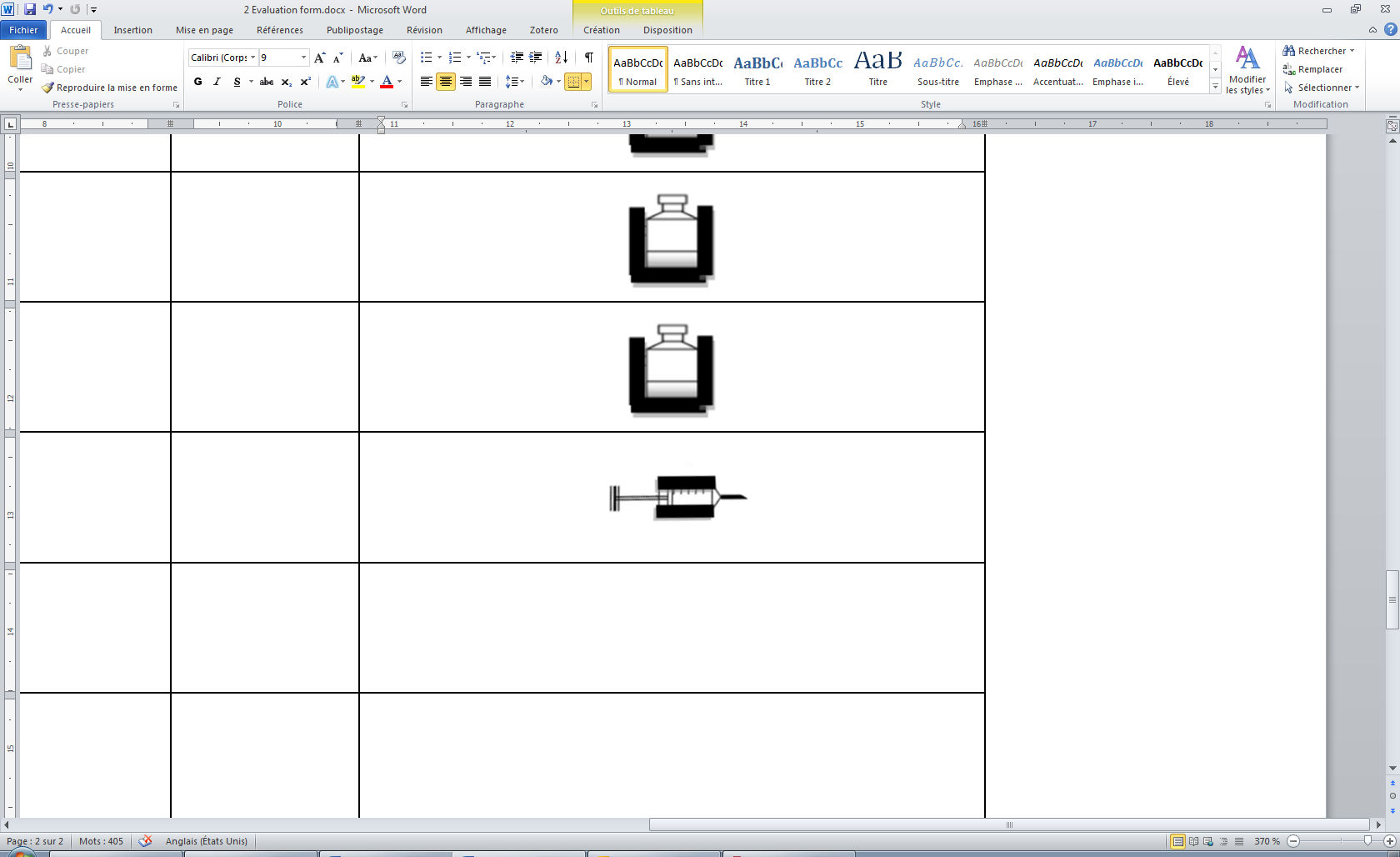 |
| Syringe S_1_ shield | - 2 | ❑ | ❑ | ❑ | 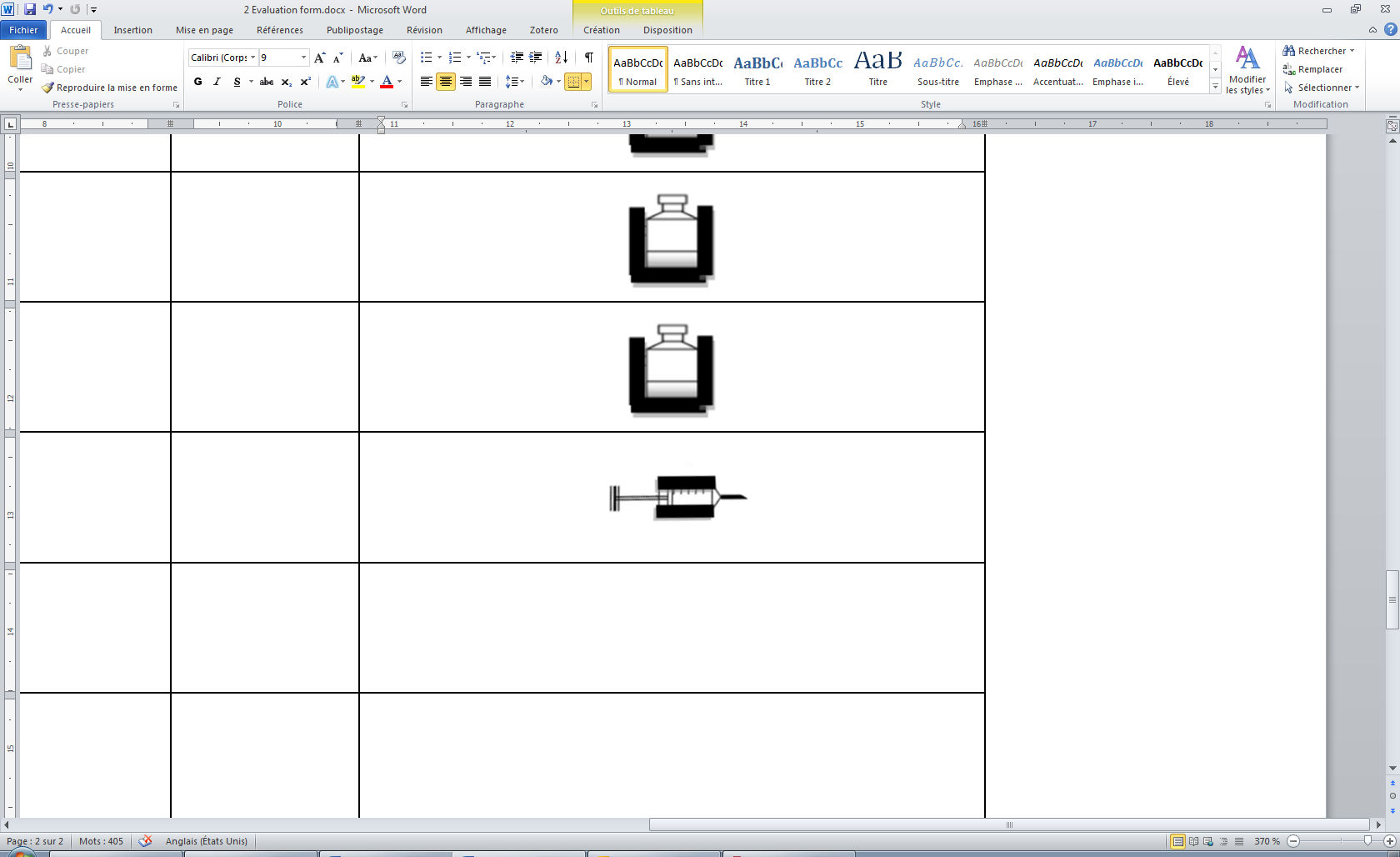 |
| Syringe S_2_ shield | - 2 | ❑ | ❑ | ❑ | 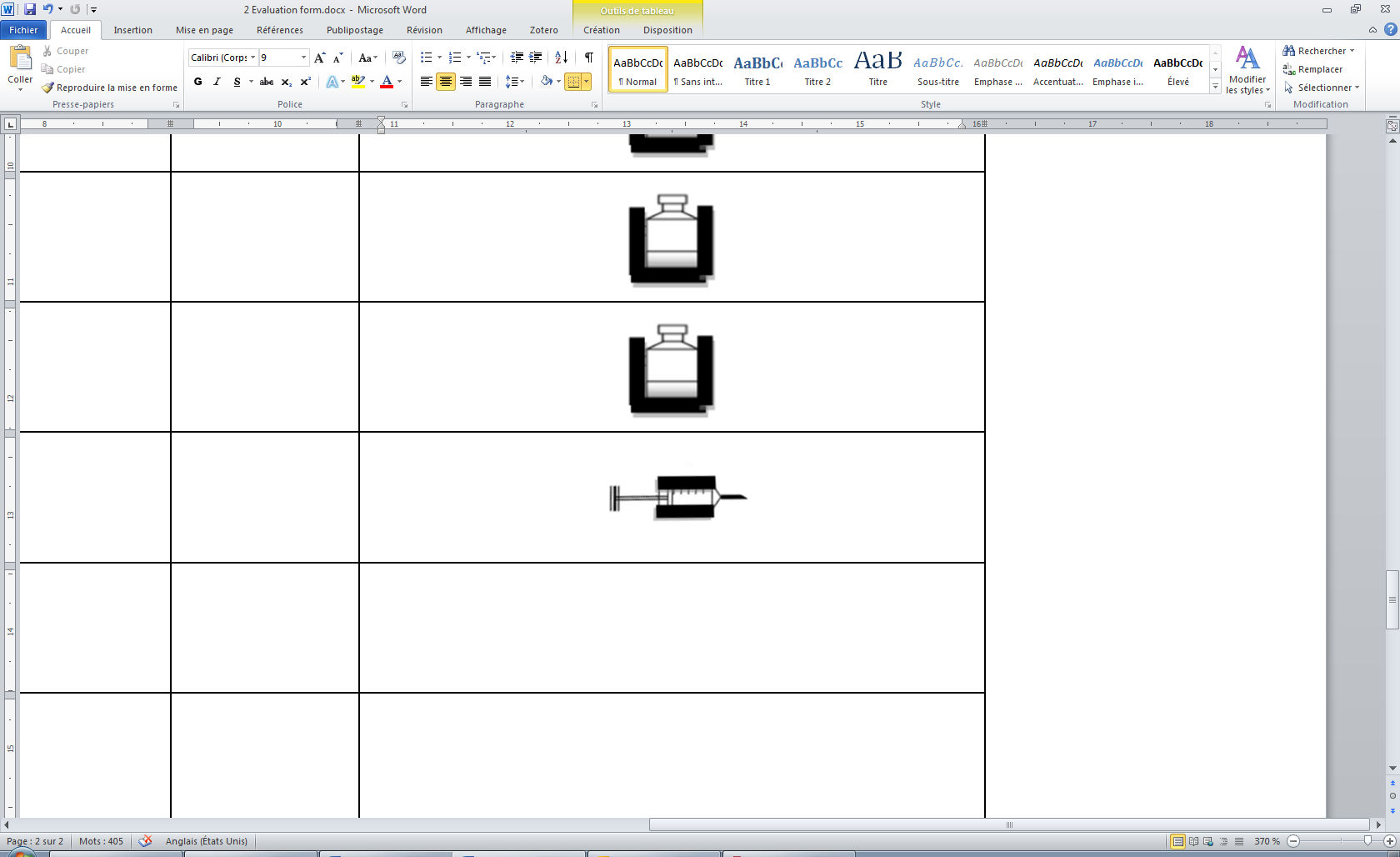 |
| Syringe S_3_ shield | - 2 | ❑ | ❑ | ❑ | 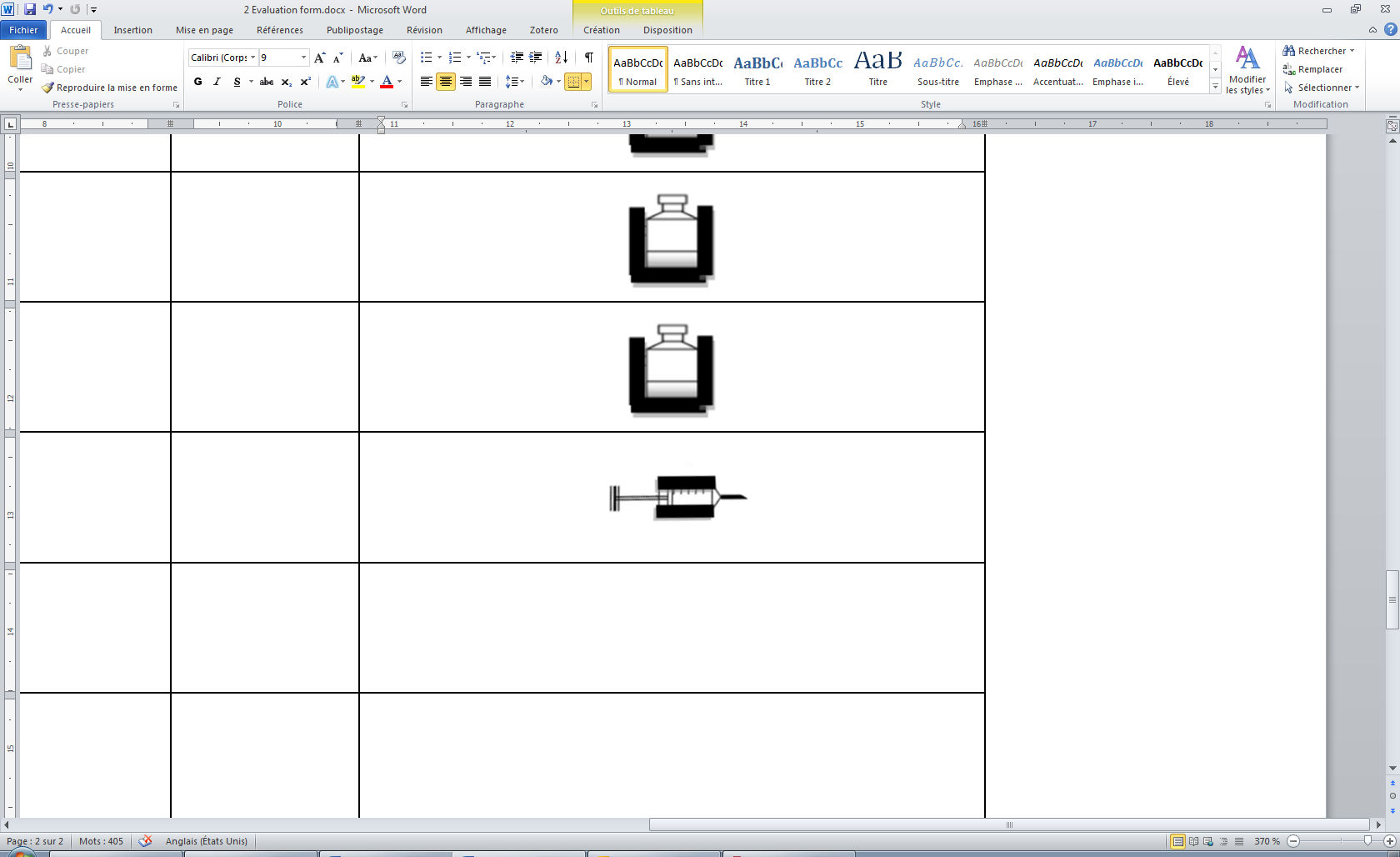 |
| Syringe S_4_ shield | - 2 | ❑ | ❑ | ❑ | 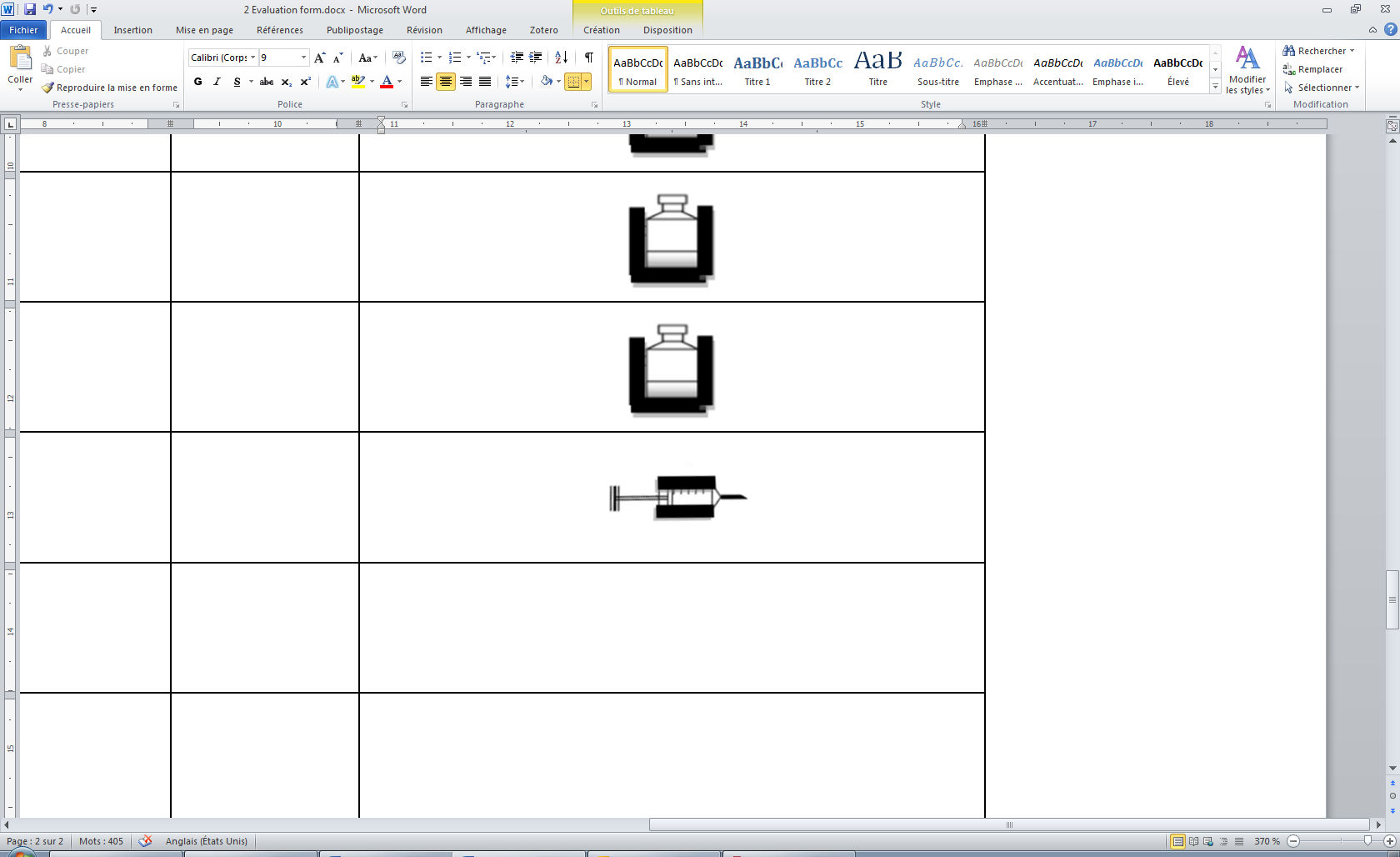 |
| Syringe S_5_ shield | - 2 | ❑ | ❑ | ❑ | 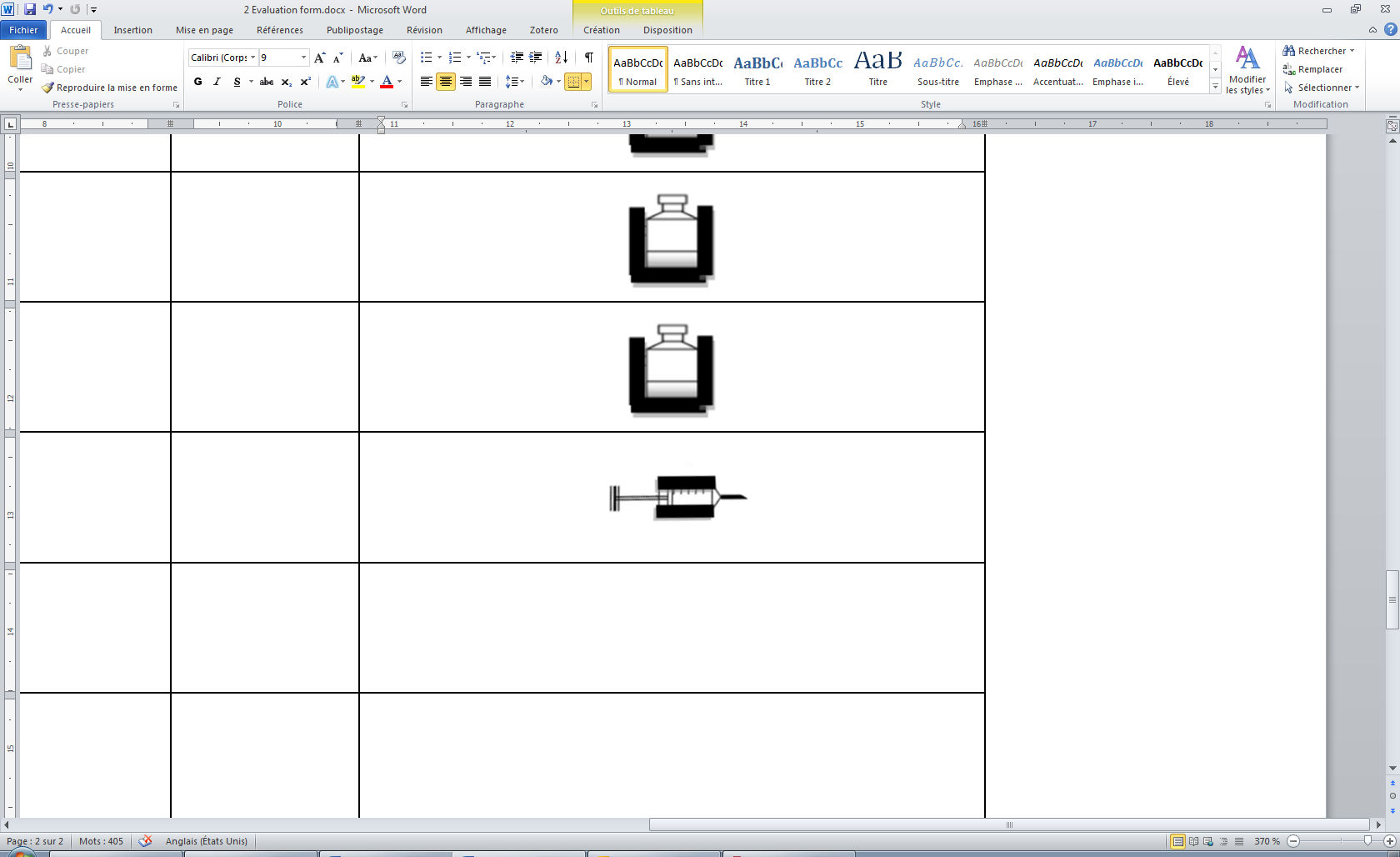 |
| Syringe S_6_ shield | - 2 | ❑ | ❑ | ❑ | 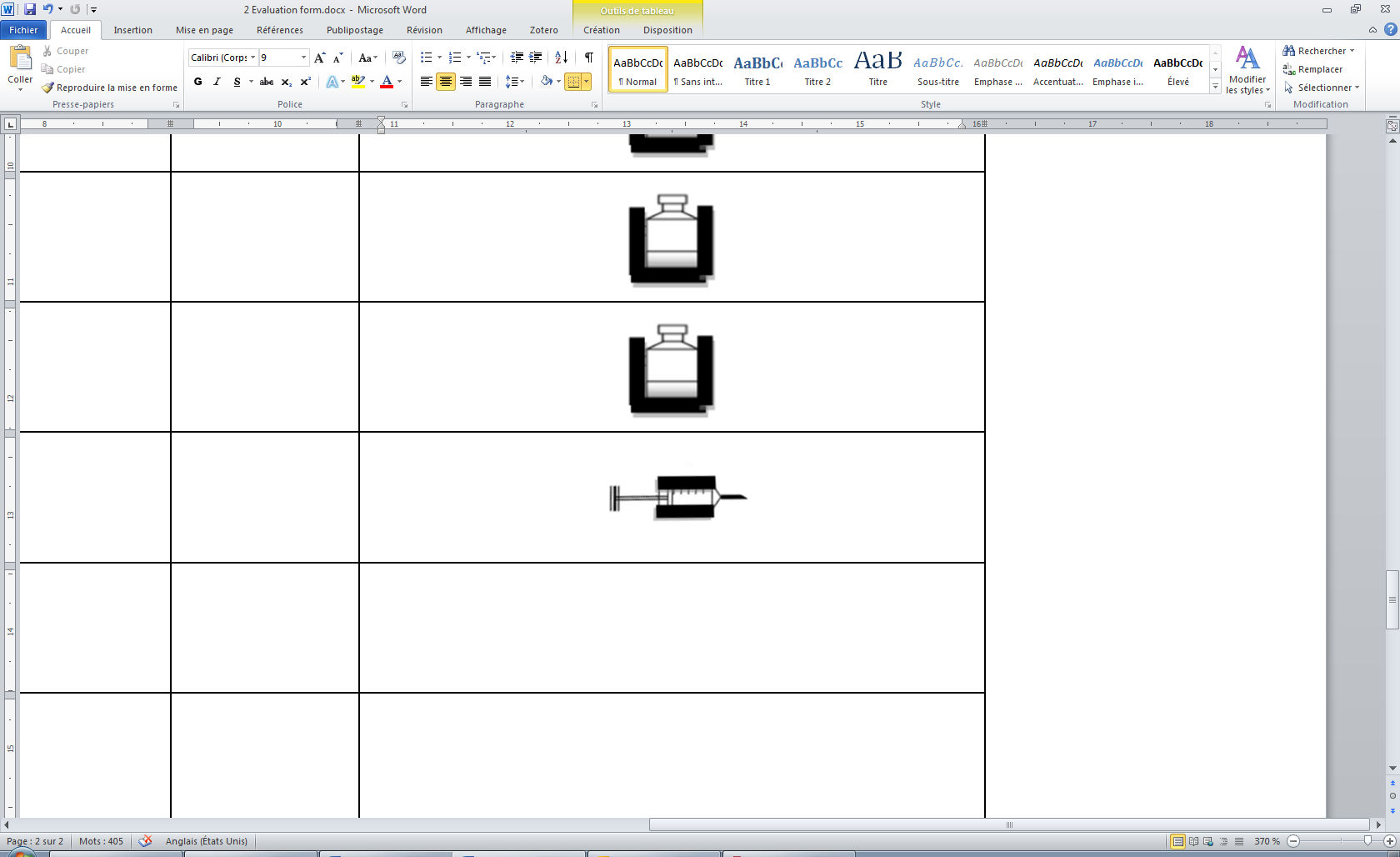 |
| **Final score** | |  | **_____/30** | |  |

Test duration: ______________ Number of fluorescence contaminations: ____________

Validation criteria: Final score > 27/30

Final score: _______/30

**Result: Fluorescent contamination evaluation ❑ Passed**

**❑ Not passed**

**Part III – Incubation monitoring**

**1) Pre- and post-evaluation bacteriological controls**

| **Sample name** | **Associated area** | **Room temperature** | | | | | | | **32 °C oven** | | | | | | |
| --- | --- | --- | --- | --- | --- | --- | --- | --- | --- | --- | --- | --- | --- | --- | --- |
|  |  | **d_1_** | **d_2_** | **d_3_** | **d_4_** | **d_5_** | **d_6_** | **d_7_** | **d_8_** | **d_9_** | **d_10_** | **d_11_** | **d_12_** | **d_13_** | **d_14_** |
|  |  |  |  |  |  |  |  |  |  |  |  |  |  |  |  |
|  |  |  |  |  |  |  |  |  |  |  |  |  |  |  |  |
|  |  |  |  |  |  |  |  |  |  |  |  |  |  |  |  |
|  |  |  |  |  |  |  |  |  |  |  |  |  |  |  |  |
|  |  |  |  |  |  |  |  |  |  |  |  |  |  |  |  |
|  |  |  |  |  |  |  |  |  |  |  |  |  |  |  |  |
|  |  |  |  |  |  |  |  |  |  |  |  |  |  |  |  |
|  |  |  |  |  |  |  |  |  |  |  |  |  |  |  |  |
|  |  |  |  |  |  |  |  |  |  |  |  |  |  |  |  |
|  |  |  |  |  |  |  |  |  |  |  |  |  |  |  |  |
|  |  |  |  |  |  |  |  |  |  |  |  |  |  |  |  |
|  |  |  |  |  |  |  |  |  |  |  |  |  |  |  |  |
|  |  |  |  |  |  |  |  |  |  |  |  |  |  |  |  |
|  |  |  |  |  |  |  |  |  |  |  |  |  |  |  |  |
|  |  |  |  |  |  |  |  |  |  |  |  |  |  |  |  |

**2) Products of the MFT-F**

**a. Original samples**

| **Sample name** | **Room temperature** | | | | | | | **32 °C oven** | | | | | | |
| --- | --- | --- | --- | --- | --- | --- | --- | --- | --- | --- | --- | --- | --- | --- |
|  | **d_1_** | **d_2_** | **d_3_** | **d_4_** | **d_5_** | **d_6_** | **d_7_** | **d_8_** | **d_9_** | **d_10_** | **d_11_** | **d_12_** | **d_13_** | **d_14_** |
| “Eluate” vial |  |  |  |  |  |  |  |  |  |  |  |  |  |  |
| Vial A |  |  |  |  |  |  |  |  |  |  |  |  |  |  |
| Vial B |  |  |  |  |  |  |  |  |  |  |  |  |  |  |
| Vial C |  |  |  |  |  |  |  |  |  |  |  |  |  |  |
| Vial D |  |  |  |  |  |  |  |  |  |  |  |  |  |  |
| Syringe S_1_ |  |  |  |  |  |  |  |  |  |  |  |  |  |  |
| Syringe S_2_ |  |  |  |  |  |  |  |  |  |  |  |  |  |  |
| Syringe S_3_ |  |  |  |  |  |  |  |  |  |  |  |  |  |  |
| Syringe S_4_ |  |  |  |  |  |  |  |  |  |  |  |  |  |  |
| Syringe S_5_ |  |  |  |  |  |  |  |  |  |  |  |  |  |  |
| Syringe S_6_ |  |  |  |  |  |  |  |  |  |  |  |  |  |  |
| Negative control vial |  |  |  |  |  |  |  |  |  |  |  |  |  |  |
| Negative control syringe |  |  |  |  |  |  |  |  |  |  |  |  |  |  |
| Positive control vial |  |  |  |  |  |  |  |  |  |  |  |  |  |  |
| Positive control syringe |  |  |  |  |  |  |  |  |  |  |  |  |  |  |
