# Supplementary material for: Implementation and validation of an in-house combined fluorescein/media-fill test to qualify radiopharmacy operators
Source: EJNMMI Radiopharm Chem. 2021 Jan 7;6:2. doi: 10.1186/s41181-020-00117-6 (PMC7790972; doi:10.1186/s41181-020-00117-6)
Supplement: Supplementary file 1 — Additional file 1. S1: TSB-F preparation protocol; S2: Operator evaluation form; S3: Detailed MFT-F protocol; S4: MFT-F cost table. [file 41181_2020_117_MOESM1_ESM.zip › Suplementary data S3.docx]

**Operators evaluation protocol**

1. In the radiopharmaceutical preparation laboratory, open an evaluation kit and carefully disinfect the package of each medical device and the septum of each vial with 70% isopropyl alcohol before entering the glovebox.
2. One vial of TSB-F mixture is used during a test, after septum disinfection.
3. Clean non-disposable equipment with a wipe soaked in a detergent-disinfectant solution. This equipment includes:

- 5 vial shields (CONT-ELU® lead container, Curium Pharma, Paris, France) identified from A to D and “Eluate”,
- Four 5 mL syringe shields (PIN-TEC™, Capintec, Florham Park, NJ, USA) identified from A to D,
- Six 3 mL syringe shields (PIN-TEC™, Capintec, Florham Park, NJ, USA) identified from 1 to 6,
- One 30 cm long forceps for unshielded vials manipulation in the glovebox.

1. Before starting the test, check the absence of fluorescent traces in the working area and on the gloves using the glovebox UV lamp.
2. Enter all the equipment in the glovebox and place a disposable sterile drape on the working area.
3. Unpack all the single-use material, prepare all the syringes and long needles with injection site, and position each vial in the corresponding lead container.
4. Identify the four 5 mL syringes as syringe A to syringe D and the six 3 mL syringes as S_1_ to S_6_.
5. The experimental filling operations are illustrated in the following figure. Two sequential 2-vials preparation simulations are performed.


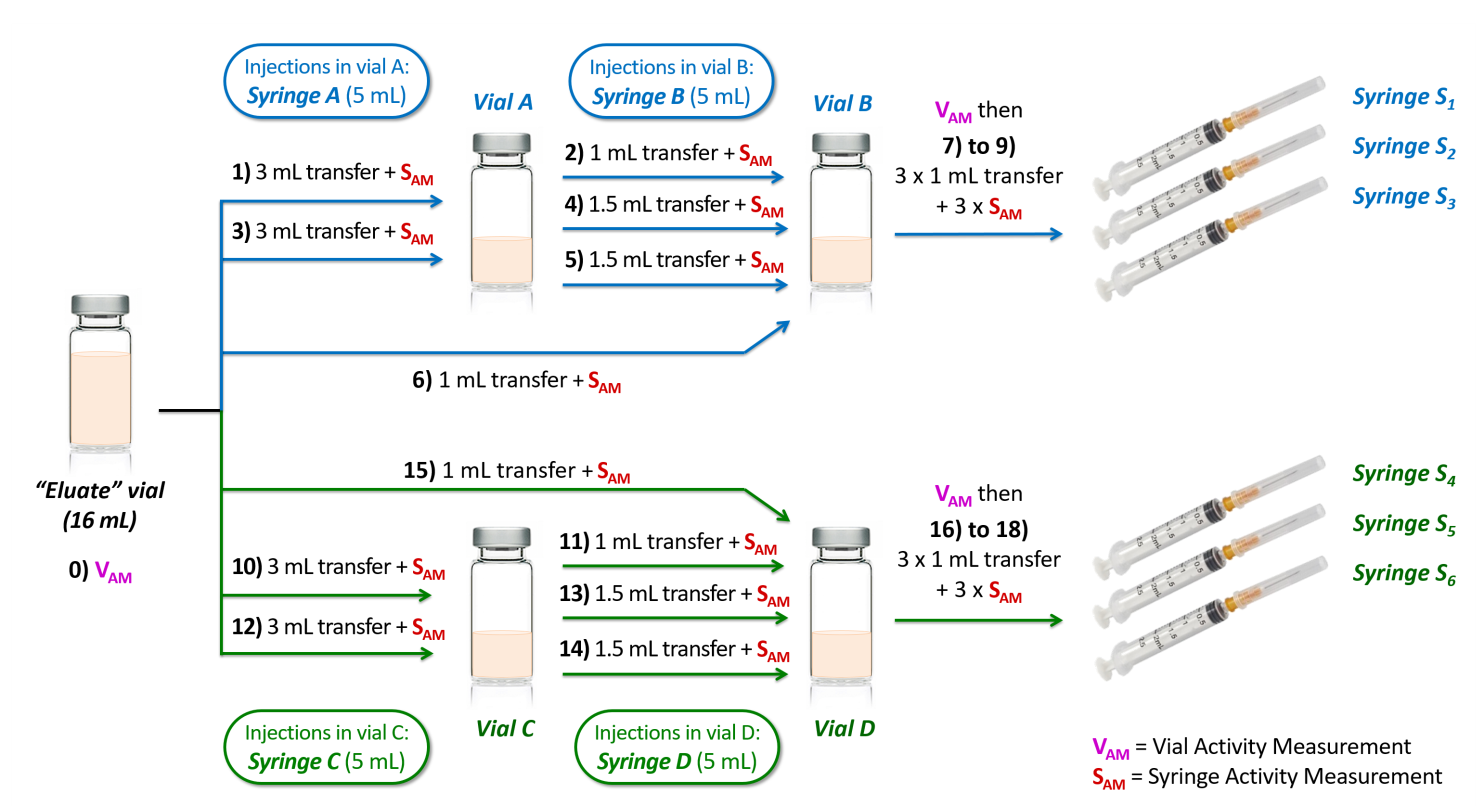


**Step-by-step instructions:**

***General notes***:

- After each withdrawal, the volume should be carefully adjusted to avoid air in the syringe, taking into account the constraints related to the syringes leaded shield.

- After each withdrawal, a measurement of syringe activity is mimicked by placing the syringe without its shield in the well of the dose calibrator.

- Before the first transfer, a measurement of “eluate” vial activity is mimicked the same way. A measurement of vials B and D activity is also simulated before the preparation of syringes S_1_, S_2_, S_3_ and S_4_, S_5_, S_6_, respectively.

1. Measure “eluate” vial activity.
2. Top “eluate” vial with a luer-lok shutter with injection site on a 23G 60 mm needle.
3. With shielded syringe A, withdraw 3 mL TSB-F from “eluate” vial, measure syringe activity and inject in vial A.
4. With shielded syringe B, withdraw 1 mL TSB-F from vial A by reverting the vial shield, measure syringe activity and inject in vial B.
5. With shielded syringe A, withdraw 3 mL TSB-F from “eluate” vial, measure syringe activity and inject in vial A.
6. Top vial A with a luer-lok shutter with injection site on a 23G 60 mm needle.
7. With shielded syringe B, withdraw 1.5 mL TSB-F from vial A, measure syringe activity and injected in vial B.
8. Repeat operation **g** once.
9. With shielded syringe B, withdraw 1 mL TSB-F from “eluate” vial, measure syringe activity and inject in vial B.
10. Measure vial B activity.
11. Top vial B with a luer-lok shutter with injection site on a 23G 60 mm needle.
12. With shielded syringe S_1_, withdraw 1 mL TSB-F from vial B and measure syringe activity.
13. Repeat operation **l** with syringes S_2_ and S_3_.
14. Leave in place the “eluate” vial and take out of the glovebox vials A and B and syringes S_1_ to S_3_, A and B.

Repeat the same sequence from operation **a** to operation **m**, replacing vial A by vial C, vial B by vial D, syringe A by syringe C, syringe B by syringe D and syringes S_1_ to S_3_ by syringes S_4_ to S_6_.
